# NMR metabolomics and glycomics for cancer detection in patients with non-specific symptoms: a prospective observational cohort study

**DOI:** 10.1016/j.ebiom.2026.106434

**Published:** 2026-08-14

**Authors:** Tereza Kacerova, Abi G. Yates, James R. Larkin, Boris Shulgin, Jack J.J.J. Miller, Philippa L. Harris Gleave, Sebastian de Jel, Jack Cheeseman, Georgia Elgood-Hunt, the SCAN Consortium, Hasan Al-Sattar, Niamh Appleby, Guanke Bao, Gagandeep Batth, Rachel Benamore, Andrew Blake, Georgeta Ciuban, Kiana K. Collins, Isabella de Vere Hunt, Helene Dreau, Monica Evans, Nick Gibb, Fergus Gleeson, Shelley Hayles, Laura Heath, Ashley Jackson, Catherine Johnson, Zoe Kaveney, David Lewis, Rosie Lomas, Daniel Mitchener, Alexandra Montagu, Julia-Ann Moreland, Niall Moore, Brian Nicholson, Sophie Roberts, Neelaksh Sadhoo, Shyamal Saujani, Anna Schuh, Andrew Slater, Sarah Smith, Nia Taylor, Sharon Tonner, Suzanne Waterfield, Louise Wing, Eric Schiffer, Daniel I.R. Spencer, Suzie Anthony, Daniel C. Anthony

**Affiliations:** a Chemistry Research Laboratory, Department of Chemistry, University of Oxford, Oxford, OX1 3TA, UK; b Kavli Institute of Nanoscience Discovery, Dorothy Crowfoot Hodgkin Building, Oxford, OX1 3QU, UK; c Physical and Theoretical Chemistry, University of Oxford, Oxford, OX1 3QZ, UK; d Department of Pharmacology, University of Oxford, Oxford, OX1 3QT, UK; e Oxford Centre for Magnetic Resonance Research, University of Oxford, Oxford, OX3 9DU, UK; f Department of Clinical Medicine, Aarhus University, Aarhus, 8200, Denmark; g numares AG, Regensburg, 93053, Germany; h Ludger Ltd, Culham Science Centre, Abingdon, OX14 3EB, UK; i SCAN Consortium, Oxford University Hospitals NHS Foundation Trust and University of Oxford, Oxford, UK; j Interventional Oncology, Department of Radiology, University of Oxford, Oxford, OX3 9DU, UK

**Keywords:** Cancer detection, Multi-omics, Metabolomics, Glycomics, Non-specific symptoms

## Abstract

**Background:**

Early cancer diagnosis in patients with non-specific symptoms is limited by the lack of discriminatory tests. Within the Oxfordshire Suspected CANcer (SCAN) pathway, exploratory biomarker work showed that serum ^1^H NMR-based metabolomics can identify cancer with high accuracy. SCAN2 evaluated whether integrating metabolomics with glycomics provides complementary molecular information and improves discrimination in a clinically complex, real-world population.

**Methods:**

Serum from 369 SCAN patients (59 cancers) was analysed using *AXINON® System*-derived NMR metabolomics and HPLC-MS glycomics. Machine-learning models were trained to predict cancer status, with performance assessed by receiver operating characteristic (ROC) analysis of pooled cross-validated predictions. To place cancer risk in a broader clinical context, a second classifier modelling alternative non-cancer diagnosis was incorporated, and mean predicted probabilities from both models were jointly projected into a two-dimensional space, maintaining strict separation of training and test data.

**Findings:**

In the full cohort, integration of glycomics with metabolomics achieved an AUC of 0.814 (95% CI 0.808–0.820). In a refined sub-cohort excluding major comorbidities and selected cancer types (32 cancers, 277 non-cancers), performance improved to an AUC of 0.884 (95% CI 0.879–0.890). Discriminatory features included cancer-associated biantennary fucosylated glycans alongside amino acid metabolites (glutamate, histidine) and lipoprotein-related measures. A classifier distinguishing metastatic from non-metastatic disease (n = 29 vs. 30) achieved an AUC of 0.80. Joint probability analysis in the full cohort preserved cancer-associated signatures across comorbidity burden, with projection-based classification achieving an accuracy of 89.2% (95% CI 85.7–92.6).

**Interpretation:**

These findings validate the SCAN1 metabolomic signature in a more clinically complex cohort and indicate that integrating glycomics with metabolomics provides complementary biological information for cancer discrimination. Joint probability analysis provides an interpretable framework for cancer risk stratification within multimorbid diagnostic pathways, supporting the clinical potential of scalable multi-omics blood testing.

**Funding:**

EPSRC, EU Horizon 2020, Wellcome/MLSTF, Novo Nordisk Foundation.

**Research in context**

**Evidence before this study**

We searched PubMed, Scopus, and Web of Science from database inception to 30 April 2026 using combinations of the terms “cancer”, “cancer detection”, “metabolomics”, “glycomics”, “multi-omics”, “blood biomarkers”, and “non-specific symptoms”. Patients presenting with non-specific symptoms represent a recognised diagnostic challenge, with a higher risk of delayed cancer diagnosis and advanced-stage disease. Blood-based metabolomic profiling has been investigated as a potential triage tool, but existing studies have largely focused on site-specific cancers or narrowly defined cohorts, limiting relevance to non-site-specific referral pathways. Evidence from real-world diagnostic populations remains limited, and independent validation under clinically realistic conditions is rarely reported. Although altered protein glycosylation is a well-established feature of malignancy, evidence for its use in patients presenting with non-specific symptoms, either alone or integrated with metabolomics, remains limited. Moreover, current approaches do not explicitly separate cancer-associated molecular signatures from those arising from general illness or comorbidity.

**Added value of this study**

This study provides independent validation of a previously identified nuclear magnetic resonance (NMR)-derived metabolic signature for cancer detection in a larger and more clinically heterogeneous cohort referred through a routine non-site-specific diagnostic pathway. We further show that integrating metabolomics with glycomics provides complementary biological information and improves discrimination between cancer and non-cancer cases. In addition, we introduce a dual-outcome machine-learning framework that separates cancer-associated molecular signals from those related to alternative non-cancer diagnoses or general illness, demonstrating stable cancer-specific signatures across a broad range of comorbidity burden. Finally, the integrated multi-omics model distinguished metastatic from non-metastatic disease, with high specificity for non-metastatic disease.

**Implications of all the available evidence**

The available evidence supports blood-based multi-omics profiling as a potential adjunct to existing screening pathways for patients presenting with non-specific symptoms. Objective molecular stratification may contribute to more consistent referral decisions, prioritisation of diagnostic investigation, and reduction of diagnostic uncertainty, while complementing clinical judgement. More broadly, these findings highlight the value of modelling cancer-specific systemic biology independently of general illness and support further prospective evaluation of scalable multi-omics blood tests in real-world primary care and rapid diagnostic settings.

## Introduction

Stage at diagnosis remains a key determinant of cancer outcomes. While current referral pathways from primary care to site-specific referral pathways perform well for patients with localising symptoms, they are less effective for individuals presenting with non-specific symptoms such as fatigue, weight loss, or persistent pain.^1^, ^2^, ^3^ For these patients, lack of a dedicated referral pathway frequently results in delayed clinical assessment and a higher likelihood of advanced disease at presentation.^3^^,^^4^

Multidisciplinary diagnostic centre (MDC) models were developed to address diagnostic gaps in patients with non-specific symptoms and have been associated with improved early cancer detection^5^ and identification of less common malignancies.^6^ In England, this approach was formalised through the Accelerate, Coordinate, Evaluate (ACE) programme and the subsequent implementation of Rapid Diagnostic Centres (RDCs).^7^ The Oxford Suspected CANcer (SCAN) pathway, initially established as an ACE Wave 2 pilot and now part of standard care, provides a real-world framework for evaluating diagnostic strategies in primary care referrals with non-specific symptoms.^8^ However, despite improved access to investigation, referral to non-site-specific pathways remains largely reliant on primary care clinical judgement, resulting in heterogeneity in referral thresholds and timing.^9^^,^^10^

Beyond imaging and routine blood tests, blood-based molecular profiling has emerged as a potential tool for earlier risk stratification in patients presenting with non-specific symptoms, reflecting the systemic metabolic impact of cancer.^11^ Metabolomic approaches have been widely applied in preclinical models and in clinically defined, site-specific malignancies, but remain underexplored in non-site-specific referral populations.^12^

In the initial SCAN study (SCAN1), blood metabolomic profiling using nuclear magnetic resonance (NMR) spectroscopy enabled discrimination between patients with non-specific symptoms with and without malignancy, and further stratified malignant cases according to metastatic status, despite substantial underlying clinical heterogeneity.^13^ Diagnostic performance was high, with sensitivities and specificities exceeding 80% for solid tumours and comparable performance for metastatic disease.^14^ A shared metabolic signature across tumour types, which was dominated by lipid-associated resonances and inflammatory glycoprotein signals, suggested systemic metabolic reprogramming rather than site-specific effects. The findings supported metabolomics as a biologically grounded triage tool within non-site-specific diagnostic pathways, complementing clinical judgement by prioritising investigation in higher-risk patients while safely excluding malignancy in others. However, limitations remained: SCAN1 was modest in size, pre-analytical handling reflected routine practice, and metabolomics alone captures only a partial host response to cancer.

Tumour-associated inflammation and altered protein glycosylation are established features of malignancy but are only partially captured by standard NMR metabolomic profiles.^15^^,^^16^ In SCAN1, the prominence of *N*-acetylated glycoprotein and sialic acid signals (GlycA/GlycB) within the cancer-associated metabolic signature indicated a contribution from inflammatory glycoprotein remodelling that could not be fully resolved by NMR alone, motivating the integration of targeted glycomic profiling to assess its complementary diagnostic value.^16^^,^^17^

We therefore hypothesised that integrating metabolomic and glycomic profiling would improve discrimination between cancer and non-cancer in patients presenting with non-specific symptoms by capturing complementary components of the systemic host response to malignancy. We further hypothesised that modelling cancer alongside alternative non-cancer diagnoses within a shared molecular feature space would enable separation of cancer-specific molecular signatures from general illness-related perturbations. The aims of this study were to validate the findings of SCAN1 in an independent cohort and to determine whether integrated metabolomic and glycomic profiling improves cancer detection and metastatic risk stratification in patients referred with non-specific symptoms.

## Methods

### Patient recruitment and eligibility criteria

Patients were recruited from the Oxfordshire Suspected CANcer (SCAN) pathway between November 2020 and July 2021, using the same non-site-specific referral framework described in the original SCAN study.^8^^,^^14^ Adults aged over forty years were eligible if referred from primary care with one or more non-specific but concerning features suggestive of cancer or other serious disease. These included unexplained weight loss, severe fatigue, persistent nausea or anorexia, new or atypical pain without a clear site-specific cause, unexplained laboratory abnormalities (e.g. anaemia, thrombocytopaenia, hypercalcaemia), or general practitioner (GP) clinical suspicion of serious disease. The latter captured cases in which the overall symptom constellation, despite being non-specific, was judged sufficiently concerning to warrant urgent investigation. Collectively, these criteria reflect the role of the SCAN pathway as a diagnostic route for patients without organ-specific symptoms but at appreciable risk of malignancy. The primary analytical model included all recruited participants. As SCAN1 demonstrated superior metabolomic discrimination for solid-organ compared with haematological malignancies, a predefined subgroup analysis in SCAN2 excluded patients with haematological cancers. To further optimise discriminatory performance, individuals with uncertain or non-biopsy-confirmed cancer diagnoses were excluded, including those with pre-malignant conditions such as Barrett's oesophagitis and monoclonal gammopathy of undetermined significance (MGUS). To minimise metabolic confounding, patients with significant renal impairment were excluded given the known effects of renal dysfunction on circulating lipid and glycoprotein profiles. Participants with prostate cancer were also excluded, as androgen-deprivation therapy substantially alters systemic metabolism and information on active or recent treatment was unavailable.

### Ethics statement

Written informed consent was obtained from all participants. The study was conducted in accordance with the UK Policy Framework for Health and Social Care Research and received ethical approval from the Oxford Radcliffe Biobank research tissue bank (20/A120). The study was conducted in accordance with the Declaration of Helsinki.

### Pre-analytical sample handling and blood collection

Blood samples for metabolomic analysis were collected immediately prior to CT imaging using standardised pre-analytical procedures.^18^ Venous blood was drawn into serum tubes without additives, following a mandated minimum fasting period of 2 h. Tubes were gently inverted and allowed to clot at ambient temperature for a standardised interval before centrifugation at 2200 g for 10 min within 30–60 min of collection. Serum was inspected for haemolysis, aliquoted into low-binding polypropylene vials, and stored at −80 °C. All collection times, processing intervals, and protocol deviations were recorded prospectively.

### NMR metabolomics acquisition

Serum aliquots were thawed at 4 °C and visually inspected for evidence of protein precipitation prior to analysis. Samples were prepared using a standardised protocol implemented by Numares AG (Regensburg, Germany), involving dilution with a deuterated phosphate buffer to ensure chemical shift stability and field locking.^19^ Serum was transferred to 5 mm NMR tubes for spectral acquisition. All ^1^H NMR experiments were performed at numares AG using a 600 MHz Bruker AVIII spectrometer. One-dimensional spectra were acquired using a presaturation-based NOESY pulse sequence to attenuate the residual water signal and enable robust detection of both low-molecular-weight metabolites and lipoprotein-associated signals. Water suppression was achieved by low-power presaturation of the water resonance during the relaxation delay and mixing period, minimising the residual water signal while preserving metabolite and lipoprotein resonances. The NOESY experiment provides robust detection of both low-molecular-weight metabolites and lipoprotein-associated signals and is widely used for quantitative serum NMR profiling. NOESY spectra were acquired with 16 scans (NS), 0 dummy scans (DS), a relaxation delay (D1) of 1.5 s, acquisition time (AQ) of 2.73 s, spectral width (SW) of 20.03 ppm, 65,536 complex data points (TD), receiver gain (RG) of 50.8, at 310 K, and a mixing time of 60 ms. In parallel, Carr-Purcell-Meiboom-Gill (CPMG) spectra were acquired using identical acquisition parameters (NS = 16, DS = 0, D1 = 1.5 s, AQ = 2.73 s, SW = 20.03 ppm, TD = 65,536, RG = 50.8, 310 K) with a spin-echo delay of 200 μs and 64 loops, corresponding to a total T2 filter of 25.6 ms. The CPMG sequence attenuates broad macromolecular and lipid signals, but does not fully suppress them, particularly in the aliphatic region (1.00–2.00 ppm), where overlap with metabolites such as branched-chain amino acids and alanine may occur.^19^^,^^20^ The CPMG experiment was acquired for untargeted metabolomics, as suppression of broad macromolecular resonances enhances small-molecule signal resolution. Targeted, quantitatively calibrated parameters were derived using the *AXINON® System* NMR platform through spectral deconvolution, including lipoprotein subclass measures (*AXINON®lipoFIT®)* as well as selected small-molecule metabolites (e.g. amino acids, myo-inositol, creatine, and creatinine). These *AXINON®lipoFIT®*-derived parameters broadly reflect particle concentrations and size-resolved lipoprotein subclasses and are similar to outputs from other NMR-based lipoprotein profiling platforms (e.g. Nightingale Health, Bruker B.I.-LISA), despite differences in subclass definitions and nomenclature. Untargeted metabolomic features were derived from the CPMG spectra, as these provide improved resolution of small-molecule signals through suppression of macromolecular contributions.

Spectra were referenced to the lactate methyl doublet (δ = 1.33 ppm). Prior to binning, spectra were aligned to correct for minor chemical shift variability arising from factors such as pH and ionic strength differences, ensuring consistent peak positions across samples and minimising bin boundary artefacts. Spectral regions between 0.55–4.25 ppm and 5.20–8.50 ppm were segmented into fixed-width bins (0.01 ppm), consistent with our previous SCAN 1 analysis, to facilitate direct comparison between studies. The resulting integrals were sum-normalised.^21^ Fixed-width binning was selected as a robust and reproducible approach for statistical analysis of the aligned spectra, providing a balance between spectral resolution and feature stability across the cohort. Untargeted metabolite assignment (SI Table S1) was supported by reference to previous experiments, literature,^18^^,^^22^ and the Human Metabolome Database.^23^

### HPLC-HRMS *N*-glycomic profiling

Serum *N*-glycan profiling was performed by Ludger Ltd. (Oxford, UK) using established and previously published workflows.^17^ *N*-glycans were enzymatically released from glycoproteins using peptide-*N*-glycosidase F (PNGase F), fluorescently labelled, and analysed by hydrophilic interaction liquid chromatography coupled to high-resolution mass spectrometry with fluorescence detection (HILIC-HRMS). Chromatographic separation was performed on a BEH-Glycan column using a Vanquish UHPLC system (Thermo Fisher Scientific), coupled to an Orbitrap Exploris 120 mass spectrometer operated in positive ion mode. System suitability standards and blanks were included throughout the analytical run to monitor performance and stability. Glycomics data were processed in Skyline software. Both fluorescence-derived chromatographic peaks, which typically represent composite glycan groups, and mass spectrometry-derived peaks corresponding to individual assigned glycans were quantified and analysed, alongside models combining both feature types. These complementary measurements capture different aspects of glycan variation, with fluorescence peaks reflecting chromatographic glycan patterns and mass spectrometry providing composition-specific assignments. Full details of sample preparation, chromatographic gradients, and mass spectrometric parameters have been described previously.^17^

### *N*-glycan nomenclature

Glycan structures were described using the standard Oxford Glycan Notation.^24^ *N*-glycans are covalently attached oligosaccharides characterised by a conserved pentasaccharide core composed of two *N*-acetylglucosamine (GlcNAc) and three mannose residues, which is subsequently elaborated through the enzymatic addition of specific monosaccharides, including fucose (**F**), additional mannose (**M**), galactose (**G**), and sialic acid (**S**), as well as by bisecting *N*-acetylglucosamine (**B**) and variations in branching patterns (antennae; **A**).^24^ Numeric descriptors indicate the number of each residue present, allowing concise representation of glycan composition (SI Table S2).

### Multivariate statistical analysis

To evaluate differences between groups, we developed an in-house analysis framework implemented in R (v4.5.2), referred to as the BTK model, using the packages *ropls* (v1.26.4), *pROC* (v1.18.0), *caret* (v6.0-94), and *randomForest* (v4.7-1.1). Missing features were addressed using sample-wise *k*-nearest neighbours (kNN) imputation. NMR data were sum normalised and the glycomics data were median normalised and log transformed prior to statistical analysis. The feature set comprised 35 NMR-derived parameters obtained using the *AXINON® System* platform (all *AXINON® System*-derived parameters are listed in SI Table S3 and mean concentrations in SI Table S4, and the complete dataset is available as SI Dataset 1), including lipoprotein subclass measures from the *AXINON®lipoFIT®* test^25^^,^^26^ and additional metabolites, including markers from the *AXINON® nephroPanel*, together with 192 NMR spectral bins (0.01 ppm resolution; individual metabolites may be represented by multiple bins corresponding to distinct resonances), 137 mass spectrometry-derived glycan features, 62 fluorescence-derived chromatographic glycan peaks, and four clinical and haematological variables (white cell count, lymphocyte count, neutrophil count, and neutrophil-to-lymphocyte ratio), yielding a total of 430 input variables. The *AXINON® System* includes parameters derived from multiple quantification modules applied to the same underlying NMR spectra. Consequently, a small number of metabolites (e.g. valine and isoleucine) are reported by both the *AXINON®lipoFIT®* and *AXINON®nephroPanel* algorithms; their agreement and influence on model performance were evaluated separately (SI Fig. S1). Feature selection was performed within cross-validation folds to mitigate redundancy and prevent over-weighting of metabolites represented by multiple features. All input features were subsequently *z*-scored to harmonise scales across measurements. Group discrimination was assessed using random forest classifiers with embedded feature selection and repeated stratified *k*-fold cross-validation. In each iteration, folds were constructed by assigning four cancer cases per fold, resulting in *k* = 14 folds (⌊59/4⌋), with class stratification preserved across splits. Model performance was evaluated using receiver operating characteristic (ROC) analysis on pooled out-of-fold predictions.^27^^,^^28^ Within each training fold, an initial random forest model was used to rank variables according to mean decrease in Gini index. The 30 highest-ranking features were retained and used to train the final classifier, which was subsequently evaluated on the corresponding held-out test fold. This feature-ranking and selection procedure was repeated independently for every cross-validation iteration using training data only. The number of retained features was chosen to reduce redundancy and model complexity while preserving the strongest discriminatory signals. The cross-validation procedure was repeated across 100 independent random permutations to assess model stability and reduce sensitivity to data partitioning. Feature selection and importance ranking were performed independently within each training fold, and class membership probabilities were generated for held-out test samples.^27^^,^^29^ ROC curves were constructed by varying the classification threshold across the full range of predicted probabilities. Sensitivity (true positive rate), specificity (true negative rate), and overall accuracy were computed at each threshold by comparing predicted class labels with observed outcomes, yielding corresponding counts of true positives (TP), true negatives (TN), false positives (FP), and false negatives (FN). The optimal classification threshold was determined using the Youden index, derived from the pooled cross-validated ROC curve. Threshold-dependent performance metrics were averaged across resampling iterations, and 95% confidence intervals were estimated from their distribution. Training and test data were separated within each iteration. Model robustness was assessed using standard label-randomisation procedures.

### BTK dual-outcome prediction framework

The BTK framework was extended to jointly model two outcomes–cancer status or alternative non-cancer diagnoses against healthy (no diagnosis)–using a shared molecular feature space. Molecular feature selection was performed exclusively within the cancer-focused subgroup defined above, and the resulting reduced feature set was fixed for all subsequent analyses. Using this shared feature space, two independent random forest models were trained: one to classify cancer vs. non-cancer, and one to distinguish alternative non-cancer diagnoses from healthy controls. Individuals not meeting the training definition for the alternative non-cancer model were excluded from model fitting, but were projected into the trained model to obtain predicted probabilities. This design ensured that both outcomes were interrogated within a common molecular feature space while preserving statistical independence between models. Cancer and alternative non-cancer diagnosis models were trained separately within each cross-validation fold, with class probabilities generated exclusively for held-out samples. For each individual, predicted probabilities were averaged across all test-fold occurrences to obtain stable estimates of cancer and alternative non-cancer diagnosis risk. Model performance was assessed using ROC analysis based on pooled cross-validated predictions. The outputs of these two models were then combined at the level of predicted probabilities (SI Fig. S2).

To examine the joint structure of these predictions, individuals were projected into a two-dimensional space defined by the mean predicted probability of cancer and the mean predicted probability of an alternative non-cancer diagnosis, represented on the logit scale.^30^^,^^31^ The distribution of samples within this joint probability space was first summarised using two-dimensional Gaussian kernel density estimation (KDE), with iso-density contours enclosing a fixed proportion of the probability mass.^30^ This provides a threshold-free description of how individuals distribute across the joint risk space. For downstream classification and performance evaluation, an explicit geometric decision region was defined within the joint probability space. A directional wedge-shaped region was derived from the cancer-associated KDE distribution to approximate the dominant axis of increasing cancer probability. Individuals falling within the wedge were classified as predicted cancer-positive, enabling construction of confusion matrices and calculation of classification metrics. To assess the robustness of this approach, the geometry of the decision boundary (a wedge-shaped region) was systematically varied across a broad parameter space, and more than 20,000 candidate configurations were evaluated. Classification performance was quantified using sensitivity, specificity, accuracy, balanced accuracy, and Youden's index, and the optimal decision boundary was selected according to overall performance. Importantly, KDE was used exclusively for descriptive visualisation of the joint probability structure, whereas the decision boundary was introduced solely to convert the continuous probability space into a discrete classification framework. All analyses were conducted with strict separation of training and test data. This strategy enables simultaneous interrogation of cancer-specific and non-cancer related molecular signals within a unified probabilistic space.

### Univariate statistical analysis

Univariate analyses were performed in R (v4.5.2) using the packages *corrplot* (v0.95), *tidyverse* (v2.0.0), *stats* (v4.1.2), and *MASS* (v7.3-58.3). Univariate associations between molecular traits and diagnostic group were assessed using odds ratios (ORs estimated from binary classification models), quantifying the change in diagnosis probability per unit increase in each feature. For each multivariable model, univariate analyses were restricted to the top 30 features identified by mean decrease in Gini index from the BTK random forest feature-ranking step. Results were visualised as forest plots with 95% confidence intervals. For continuous variables, group differences were evaluated using unpaired two-sample t tests, and for categorical variables using the chi-square test. All resulting p-values were adjusted for multiple testing using false discovery rate correction (Benjamini Hochberg, FDR <0.05). Correlations among significantly altered molecular features were assessed using Pearson correlation analysis. To assess robustness to the choice of correlation metric, the analysis was repeated using Spearman rank correlations, yielding comparable results (SI Fig. S3). Data are presented as Tukey boxplots with whiskers extending to 1.5× the interquartile range (GraphPad Prism 10).

### Role of funders

The study was supported by the funding sources listed in the Acknowledgements. numares AG provided in kind support for NMR metabolomics data acquisition and technical data processing. Ludger Ltd performed the glycomics analyses on a fee for service basis and carried out technical processing of the glycomics data. Apart from the contributions of company employees who fulfilled the criteria for authorship, the funding organisations had no role in study design, data analysis, interpretation of the results, manuscript preparation, or the decision to submit the manuscript. No intellectual property arose from this work.

## Results

### Cohort characteristics and application of eligibility criteria

The SCAN2 cohort comprised 369 primary care referrals with non-specific symptoms, of whom 59 were diagnosed with cancer and 310 had malignancy excluded. Recruitment was conducted under stricter pre-analytical conditions than SCAN1, including standardised fasting and immediate serum separation, to reduce technical variability. Despite these refinements, the cohort remained clinically heterogeneous, with diverse tumour sites and multiple comorbidities across diagnostic group.

**Fig. 1:**
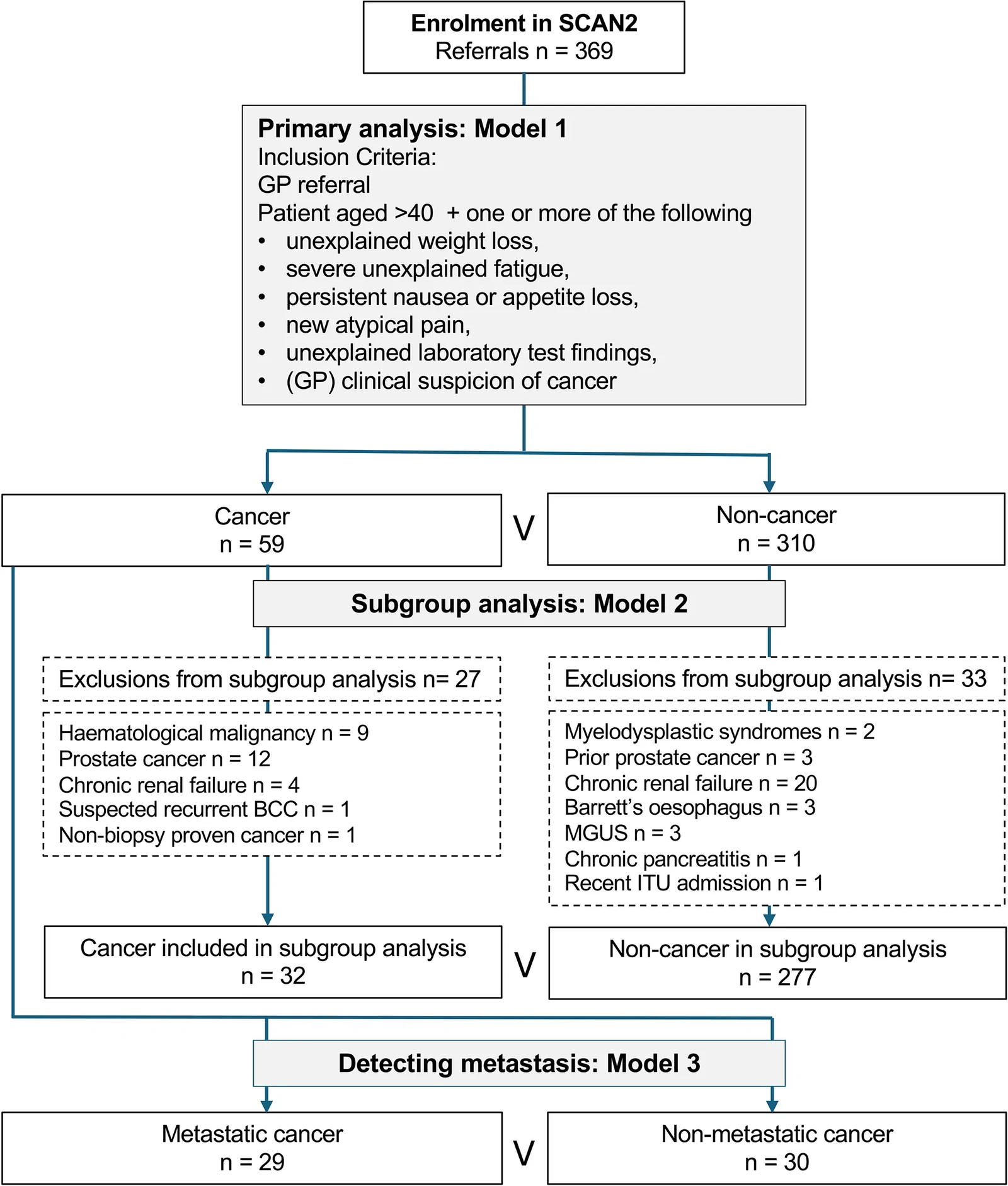
**Overview of the SCAN2 cohort and study inclusion criteria.** Flow diagram summarising participant selection from the SCAN pathway, key inclusion and exclusion criteria, and subgroup definitions used for the model comparisons. The cancers and non-cancers excluded from the subgroup analysis were those participants removed according to predefined criteria based on the SCAN1 results, including haematological malignancies, non-biopsy-confirmed or pre-malignant diagnoses, prostate cancer, or significant renal impairment. MGUS: Monoclonal gammopathy of undetermined significance, BCC: basal cell carcinoma, ITU: intensive therapy unit.

Three complementary analytical models were constructed (Fig. 1). The primary model included all participants (n = 369) and assessed overall discrimination between cancer (n = 59) and non-cancer cases (n = 310). The second model specifically targeted solid-organ cancers, excluding patients with haematological malignancies. In this subgroup analysis, participants were excluded due to potential metabolic confounding, including haematological malignancies, non-biopsy-confirmed or pre-malignant diagnoses, prostate cancer, and significant renal impairment (Fig. 1). This group comprised 32 cancer cases and 277 non-cancer participants (Fig. 1). Finally, to examine whether metabolic and glycomic signatures varied with disease burden, a third model stratified all cancer cases in the cohort into metastatic (n = 29) and non-metastatic (n = 30) groups. Since detailed TNM staging was not uniformly available across tumour types, metastatic status was used as a clinically meaningful surrogate of disease stage.

Demographic and clinical characteristics of participants across the three analytical models are summarised in Table 1. The SCAN2 cohort comprised 369 individuals (59 cancer, 310 non-cancer), with a broad range of cancer types represented, including gastrointestinal, lung, urogenital, and haematological malignancies (SI Table S5). This heterogeneity reflects the non-site-specific diagnostic setting of the study. Across the entire cohort, participants with cancer were significantly older than non-cancer participants (74.7 ± 10.1 vs. 69.5 ± 13.0 years; p = 0.004), whereas sex distribution and body mass index (BMI) did not differ between groups. A similar age difference was observed in the rigorously stratified subgroup model (75.0 ± 10.8 vs. 68.3 ± 12.9 years; p = 0.005), again with no significant differences in sex or BMI. In contrast, no significant differences in age, sex, or BMI were observed between metastatic and non-metastatic cancer cases. Sensitivity analyses performed separately in male and female subclasses showed comparable model performance, indicating that the observed signal is consistent across sexes (SI Figs. S4 and S5, SI Table S6).

**Table 1:** Demographic and clinical characteristics of patients across the three classification models (all SCAN2 participants, rigorously stratified sub-cohort, and metastatic cancer model).

Characteristics	Non-cancer	Cancer	p*-*value
n (participants)	310	59	
Age at SCAN	69.5 ± 13.0	74.7 ± 10.1	0.004
Sex (% female)	167 (54%)	28 (47%)	0.395
Body mass index, kg m^−2^	26.2 ± 5.3	26.2 ± 5.8	0.998
n (refined cohort participants)	277	32	
Age at SCAN	68.3 ± 12.9	75.0 ± 10.8	0.005
Sex (% female)	151 (55%)	22 (69%)	0.136
Body mass index, kg m^−2^	26.2 ± 5.3	24.8 ± 4.7	0.170
	**Non-metastatic**	**Metastatic**	

n (participants)	30	29	
Age at SCAN	73.9 ± 10.2	75.6 ± 9.8	0.537
Sex (% female)	13 (43%)	15 (52%)	0.606
Body mass index, kg m^−2^	26.4 ± 6.9	26.0 ± 4.3	0.815

Data are presented as mean ± standard deviation. Between-group comparisons were performed using unpaired two-tailed t-tests for continuous variables and chi-square tests for categorical variables. A p-value <0.05 was considered statistically significant. BMI data were available for 340 of 369 patients in the full cohort, for 288 of 309 patients in the rigorously stratified model, and for 54 of 59 patients in the metastatic cancer model.

The observed age difference between cancer and non-cancer participants likely reflects the larger and more heterogeneous non-cancer cohort. To evaluate the impact of age on model performance, classification models were constructed with and without age as a covariate. Inclusion of age yielded an ROC AUC of 0.814 (95% CI 0.808–0.820), while exclusion resulted in a comparable AUC of 0.815 (95% CI 0.809–0.821), indicating minimal influence of age on discrimination. Consistent with this, age ranked 258th in variables by feature importance and was not included in the primary predictive signature. Correlation analyses between age and top-ranked features showed uniformly weak and dispersed associations (|r| < 0.35), confirming that model performance was driven by metabolic and glycomic features rather than age-related effects (SI Figs. S6 and S9). Furthermore, analyses in an age-matched subset yielded comparable model performance (SI Fig. S10, SI Table S7). Additionally, estimated glomerular filtration rate (eGFR), determined from serum creatinine levels, showed a modest univariable association with cancer status (p = 0.013, SI Fig. S11A), but it was not prioritised in multivariable modelling, ranking 106th in variables by feature importance, and its inclusion did not materially affect model performance (ROC AUC 0.809 (95% CI 0.803–0.815)), indicating that residual renal function differences did not drive discrimination (SI Fig. S11B).

### Replication of the SCAN1 metabolomic signature in a more heterogeneous cohort

We previously reported that serum metabolomic profiling robustly discriminated cancer from non-cancer participants, and metastatic from non-metastatic disease, in the SCAN1 cohort. To evaluate the generalisability of these findings under increased clinical heterogeneity, we performed independent metabolomic profiling and model development in the SCAN2 cohort (n = 369; 59 cancers, 310 non-cancers) using a different analytical NMR FDA-cleared platform and modelling strategy.

In SCAN2, multivariable modelling identified nine NMR-derived metabolomic features (glutamate, glutamine, histidine, myo-inositol, an overlapping valine/proline signal, alanine, lactate, sialic acid signal (GlycB), and high-density lipoproteins (HDLs)) that were able to discriminate cancer from non-cancer participants with a ROC AUC of 0.783 (95% CI 0.774–0.791) (SI Fig. S12A). Predicted probability distributions showed clear separation between diagnostic groups, indicating preservation of classification structure despite substantial cohort heterogeneity (SI Fig. S12B).

To assess external validity and exclude cohort- or platform-specific effects, the SCAN2-derived metabolite panel was then applied to the previously published SCAN1 cohort. This reciprocal validation yielded stronger discrimination (AUC 0.881 (95% CI 0.875–0.896); SI Fig. S12C), with predicted vs. observed probabilities confirming robust separation between cancer and non-cancer participants (SI Fig. S12D). Univariate odds ratios derived from logistic regression models were directionally concordant (SI Fig. S12E), demonstrating consistency of individual metabolite–disease associations.

Together, these findings demonstrate bidirectional replication across temporally independent cohorts, analytical platforms, and modelling strategies. Discriminatory metabolic structure originally reported in SCAN1 was reproduced in the more heterogeneous SCAN2 cohort, while metabolites independently prioritised in SCAN2 retained both discriminatory performance and effect direction when re-evaluated in the previously published SCAN1 dataset. This convergence provides strong evidence for the robustness and generalisability of the NMR-derived metabolic signature in non-specific symptom populations.

### NMR metabolomics and LC-MS glycomics detect cancer in a cohort with non-specific symptoms

**Fig. 2:**
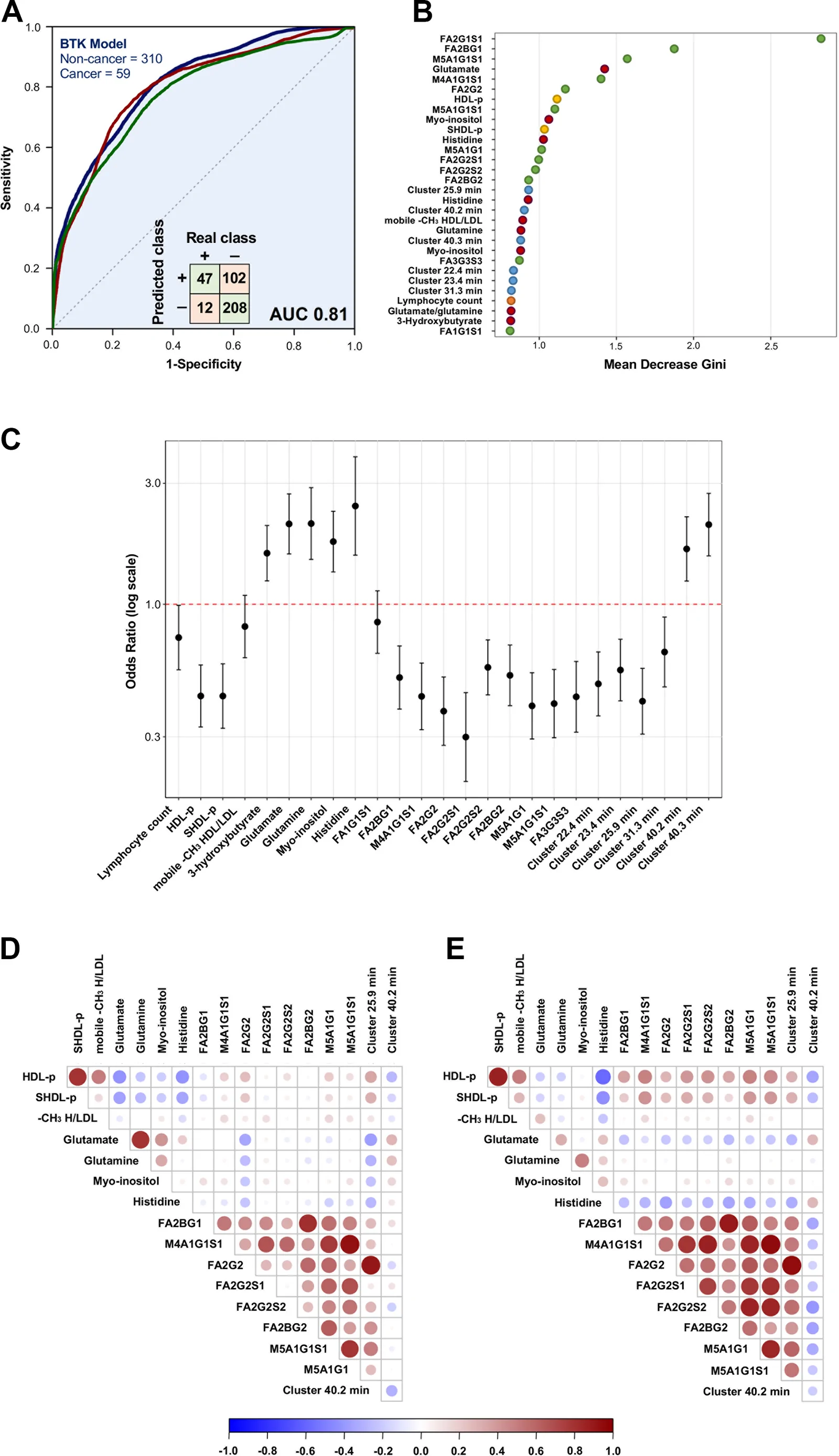
**Integrated metabolomic and glycomic analysis of cancer and non-cancer participants. (A)** Receiver operating characteristic (ROC) curves for the integrated model (blue), NMR-only model (red), and glycomics-only model (green). The inset shows the confusion matrix at the selected decision threshold. **(B)** Variable importance plot showing the top features ranked by mean decrease in Gini from the Random Forest classifier, including NMR-derived metabolites, lipoprotein measures, and glycan features. **(C)** Odds ratios (log scale) with 95% confidence intervals for the principal discriminatory variables estimated using logistic regression. **(D)** Pearson correlation matrix of the selected metabolites, lipoprotein measures, and glycan features in non-cancer participants. **(E)** Pearson correlation matrix of the same variables in cancer participants. Positive correlations are shown in red and negative correlations in blue; circle size and colour intensity indicate the magnitude of the correlation coefficient. HDL-p, high-density lipoprotein particle concentration; SHDL-p, small high-density lipoprotein particle concentration.

**Fig. 3:**
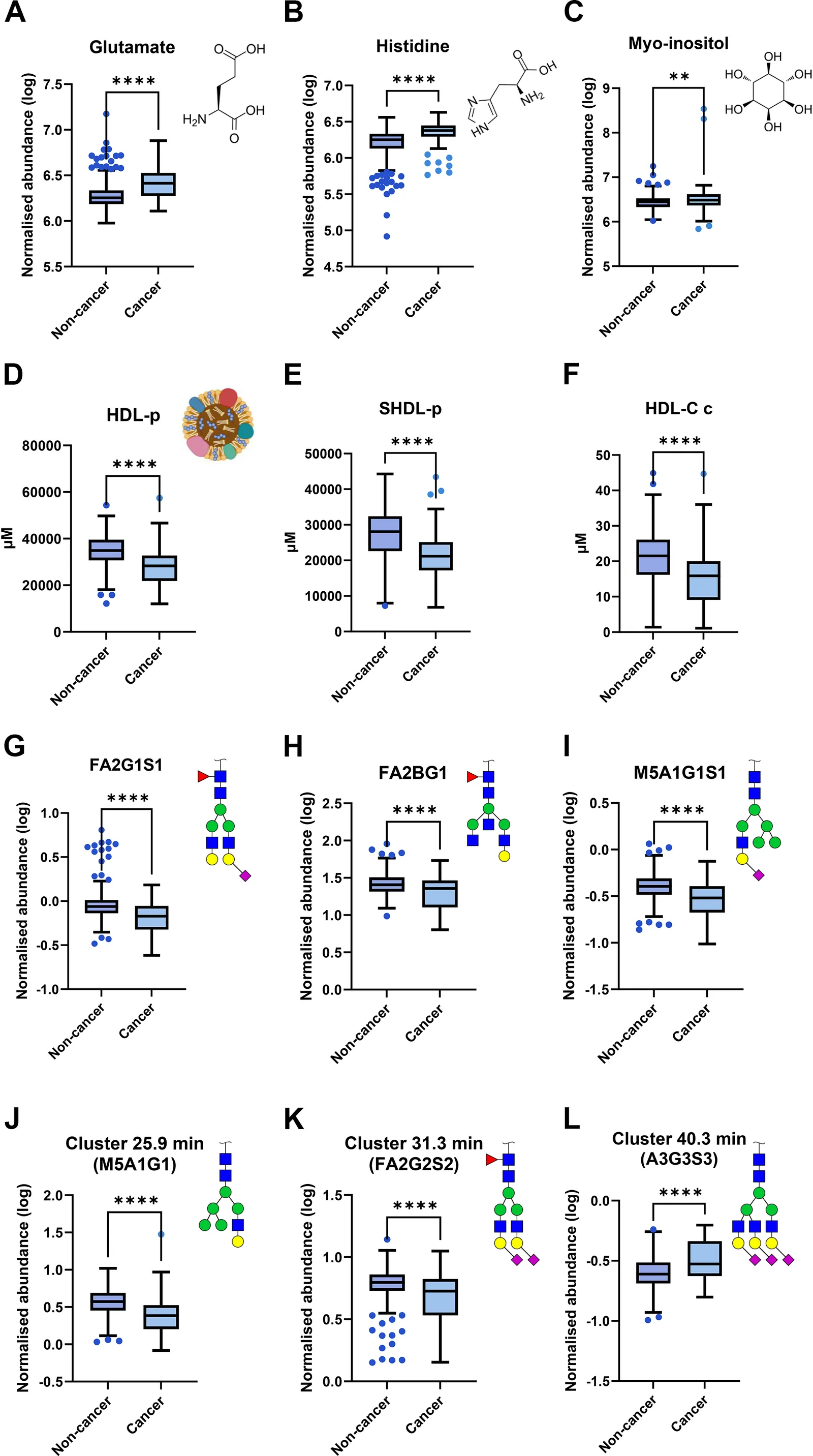
**Selected metabolomic, lipoprotein, and glycomic features in cancer and non-cancer participants (n = 369; 59 cancer, 310 non-cancer).** Boxplots show the distribution of selected metabolites, lipoprotein measures, glycans, and glycan clusters in cancer and non-cancer participants. **(A)** Glutamate, **(B)** Histidine, **(C)** Myo-inositol, **(D)** HDL particle concentration (HDL-p), **(E)** Small HDL particle concentration (SHDL-p), and **(F)** HDL cholesterol concentration (HDL-C c) were selected from the integrated NMR model based on Random Forest variable importance (mean decrease Gini). **(G)** FA2G1S1, **(H)** FA2BG1, and **(I)** M5A1G1S1 were selected from the glycomics-only model integrating fluorescence and mass spectrometry data (SI Table S2). **(J)** Cluster 25.9 min (predominantly M5A1G1), **(K)** Cluster 31.3 min (predominantly FA2G2S2), and **(L)** Cluster 40.3 min (predominantly A3G3S3) correspond to fluorescence-derived glycan clusters. Statistical significance was assessed using unpaired two-sided *t*-tests with false discovery rate (FDR) correction for multiple testing. Asterisks indicate significance (∗∗p < 0.01; ∗∗∗∗p < 0.0001).

To enhance performance in the SCAN2 cohort and better capture lipid-related metabolic perturbations, the NMR-only framework was extended to incorporate deconvoluted, lipoprotein-focused NMR panels, enabling refined characterisation of HDL subfractions and particle distributions beyond conventional spectral measures.^19^ In addition to metabolomic features, basic clinical and demographic variables and routine haematological parameters, including white cell count, lymphocyte count, neutrophil count, and the neutrophil-to-lymphocyte ratio, were evaluated. These variables did not rank among the dominant predictors and did not materially influence model performance. The combined untargeted NMR metabolome and targeted NMR-derived parameters (*AXINON® System,* SI Table S4), including lipoprotein subclass measures and lipid-associated signals (e.g. HDL-derived components), retained strong discriminatory capacity, replicating findings from SCAN1. The model achieved an AUC of 0.799 (95% CI 0.793–0.805) (Fig. 2A, red; SI Fig. S13A), with an accuracy of 76.7% (95% CI 75.6–77.6), sensitivity of 71.7% (95% CI 70.9–72.4), and specificity of 77.7% (95% CI 77.2–78.2), closely matching performance in SCAN1 (AUC 0.832 (95% CI 0.721–0.951)).^14^ Clear separation between cancer and non-cancer cases was observed despite increased clinical heterogeneity (Fig. 2A). Notably, these signatures were consistent across multiple tumour types, supporting their interpretation as a generalisable host-response signal rather than tumour-specific metabolic effects. Top-ranked NMR variables included glutamate (p < 0.0001), histidine (p < 0.0001), and myo-inositol (p = 0.003), which were elevated in cancer, alongside reductions in lipoprotein measures, including HDL particle concentration (HDL-p; p < 0.0001), small HDL particles (SHDL-p; p < 0.0001), and HDL cholesterol (HDL-C; p < 0.0001) (Fig. 3A–F). Odds ratio analysis (SI Table S8; Fig. 2C) demonstrated consistent pathway-level differences, with higher glutamate (OR 2.08 (95% CI 1.58–2.73)), myo-inositol (OR 1.77 (95% CI 1.34–2.33)), and histidine (OR 2.44 (95% CI 1.56–3.82)), and lower HDL-p concentrations (OR 0.44 (95% CI 0.33–0.58)) in cancer.

To explore complementary molecular mechanisms, semi-targeted glycomic profiling was evaluated. Glycan features alone provided moderate discrimination between cancer and non-cancer cases, with a ROC AUC of 0.778 (95% CI 0.771–0.785). At the decision threshold (0.141), the model achieved a sensitivity of 76.0% (95% CI 75.2–76.8) and a specificity of 66.9% (95% CI 65.9–68.0), corresponding to an overall accuracy of 68.3% (95% CI 67.7–68.9) (Fig. 2A, green; SI Fig. S13B). Although less discriminatory than the NMR-derived metabolomic profile, glycomic features captured biologically informative alterations associated with malignancy. The most discriminatory glycans included FA2G2S1 (OR 0.30 (95% CI 0.20–0.45)), FA2BG1 (OR 0.51 (95% CI 0.39–0.69)), and M5A1G1S1 (OR 0.41 (95% CI 0.30–0.55)), all fucosylated or bisected biantennary structures reduced in cancer (p < 0.0001; Fig. 3G–I). Consistent decreases were also observed for fluorescence-detected peaks corresponding predominantly to M5A1G1 (OR 0.42 (95% CI 0.31–0.56)) and FA2G2S2 (OR 0.65 (95% CI 0.47–0.89)) (both p < 0.0001; Fig. 3J). In contrast, higher-order glycans, notably A3G3S3, were increased in cancer (OR 2.06 (95% CI 1.55–2.74); p < 0.0001; Fig. 3L). The direction and magnitude of glycomic changes were coherent across platforms and inversely related to NMR-derived metabolic alterations, with reduced fucosylated and bisected structures parallelling higher glutamate, histidine, and myo-inositol, and lower HDL-p and SHDL-p concentrations. Integration of untargeted NMR metabolomics, targeted AXINON® measurements and semi-targeted glycomic data produced a numerically higher ROC AUC of 0.814 (95% CI 0.808–0.820) compared with the metabolomics-only model (AUC 0.799). However, this difference was not statistically significant (DeLong p = 0.328). The combined model achieved a ROC AUC of 0.814 (95% CI 0.808–0.820; Fig. 2A, Table 2). At the decision threshold (0.129), sensitivity reached 80.3% (95% CI 79.1–81.5) and specificity 67.0% (95% CI 66.1–67.9), corresponding to an overall accuracy of 70.0% (95% CI 69.1–70.9). Examination of the associated confusion matrix (47 TP, 102 FP, 208 TN, and 12 FN) indicates that the operating point represents a balanced compromise between maximising cancer detection and limiting false classifications. Although the integrated model was not significantly different from the metabolomics-only model (AUC 0.814 vs. 0.799; DeLong p = 0.328), discriminatory features were retained from all modalities (Fig. 2B), with fucosylated and bisected glycans (FA2G2S1 (p < 0.0001, Fig. 3G), FA2BG1 (p < 0.0001, Fig. 3H), M5A1G1S1 (p < 0.0001, Fig. 3I)) ranking alongside NMR-derived metabolites (glutamate (p < 0.0001, Fig. 3A), histidine (p < 0.0001, Fig. 3B), myo-inositol (p = 0.003, Fig. 3C)) and HDL subclasses (HDL-p, SHDL-p, HDL-C (all p < 0.0001, Fig. 3D–F)). In addition, fluorescence- and MS-derived glycan clusters capturing related structural motifs (M5A1G1, FA2G2S2, and A3G3S3; Fig. 3J–L) were retained, indicating that both individual glycan species and higher-order glycan patterns contribute to model discrimination. Odds ratio analysis confirmed opposing trends across modalities, with higher cancer risk associated with elevated amino acid metabolites and lower risk linked to reduced HDL measures and fucosylated glycans. Although glycomics alone showed lower discriminatory performance, the retained glycan features indicate that glycomics provides complementary biological information alongside metabolomic profiling.

**Table 2:** Diagnostic performance of metabolomic, glycomic, and integrated models in SCAN2.

Model	AUC	AUC (95% CI)	Accuracy (95% CI)	Sensitivity (95% CI)	Specificity (95% CI)	*n* case	*n* control
NMR metabolomics (binned and deconvoluted data)	0.799	0.793–0.805	76.7% (75.6–77.6)	71.7% (70.9–72.4)	77.7% (77.2–78.2)	59	310
LC-MS glycomics	0.778	0.771–0.785	68.3% (67.7–68.9)	76.0% (75.2–76.8)	66.9% (65.9–68.0)	59	310
Integrated multi-omics model (NMR + glycomics)	0.814	0.808–0.820	70.0% (69.1–70.9)	80.3% (79.1–81.5)	67.0% (66.1–67.9)	59	310
Strict selection (multiomics)	0.884	0.879–0.890	77.5% (77.0–77.9)	86.0% (84.8–87.2)	76.5% (76.0–77.0)	32	277
Metastatic classifier (multi-omics)	0.801	0.789–0.813	75.2% (72.6–77.7)	59.4% (57.6–61.2)	90.9% (89.0–92.8)	29	30

Performance metrics are reported for classification of cancer vs. non-cancer (n cases = number of patients with cancer; n controls = number of patients without cancer) and for metastatic vs. non-metastatic disease (n cases = number of patients with metastatic cancer; n controls = number of patients with non-metastatic cancer).

Finally, to characterise relationships between individual metabolites and glycans, Pearson correlation analyses were performed separately in non-cancer and cancer groups (Fig. 2D and E). In both groups, NMR-derived metabolites (upper left quadrant) formed a distinct cluster from glycan features (lower right quadrant), indicating minimal overlap between these biochemical domains. In non-cancer samples (Fig. 2D), positive correlations were observed primarily within each modality, including among amino acids (glutamate, histidine, myo-inositol) and among structurally related glycans (FA2G2S1, FA2BG1, M5A1G1S1). In contrast, cancer samples (Fig. 2E) showed a reorganisation of correlation structure, characterised by stronger inverse associations between histidine and fucosylated and bisected glycans, alongside pronounced positive correlations between glycan signals and HDL-related measures (HDL-p, SHDL-p, and mobile lipoprotein –CH_3_ signals). This pattern reflects a coordinated shift in metabolic connectivity, with altered coupling between lipid and glycan metabolism emerging specifically in malignancy. Together, these findings demonstrate that the NMR metabolome provides a reproducible diagnostic signal, with glycomic alterations contributing complementary discriminatory information. Importantly, this disease-associated signal remained robust despite substantial clinical heterogeneity, supporting its potential applicability in diverse real-world settings and providing a foundation for subsequent refined and stage-specific analyses.

### Enhanced discrimination after exclusion of major non-malignant metabolic confounders

**Fig. 4:**
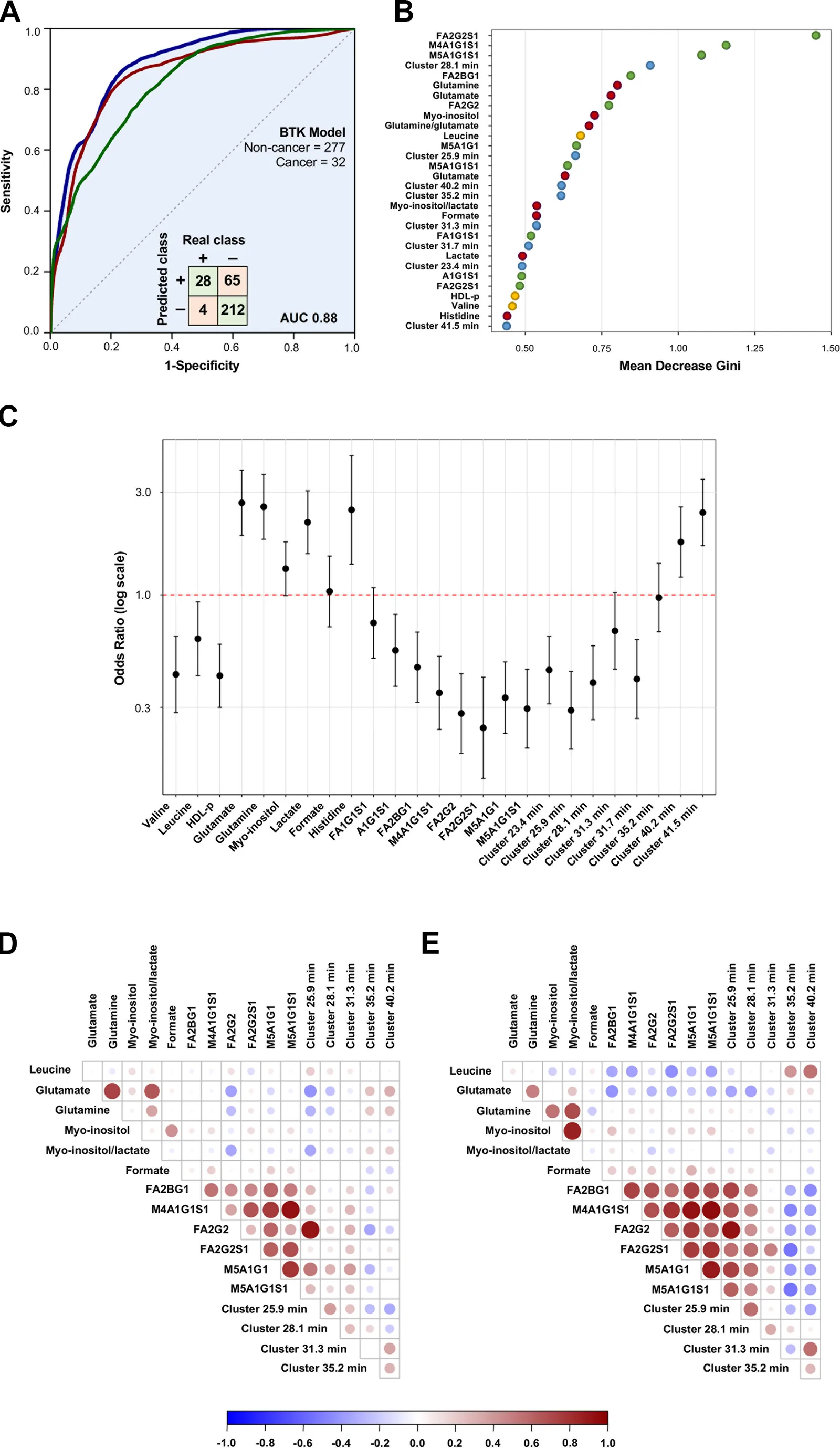
**Integrated metabolomic and glycomic analysis of the refined cohort after exclusion of major metabolic confounders (n = 309; 32 cancer, 277 non-cancer). (A)** Receiver operating characteristic (ROC) curves for the integrated model (blue), NMR-only model (red), and glycomics-only model (green). The inset shows the confusion matrix at the selected decision threshold. **(B)** Variable importance plot showing the top features ranked by mean decrease in Gini from the Random Forest classifier, including NMR-derived metabolites, lipoprotein measures, and glycan features (SI Table S2). **(C)** Odds ratios (log scale) with 95% confidence intervals for the principal variables estimated using logistic regression. **(D)** Pearson correlation matrix of the selected metabolites, lipoprotein measures, and glycan features in non-cancer participants. **(E)** Pearson correlation matrix of the same variables in cancer participants. Positive correlations are shown in red and negative correlations in blue; circle size and colour intensity indicate the magnitude of the correlation coefficient.

To minimise metabolic confounding from non-malignant conditions, we evaluated a refined subgroup model (277 non-cancer; 32 cancer). In this cohort, the BTK model demonstrated significantly enhanced discriminatory performance (p < 0.0001) compared with the full-cohort model, achieving an AUC of 0.884 (95% CI 0.879–0.890), with accuracy, sensitivity, and specificity of 77.5% (95% CI 77.0–77.9), 86.0% (95% CI 84.8–87.2), and 76.5% (95% CI 76.0–77.0), respectively (Fig. 4A; Table 2). The corresponding confusion matrix (TP = 28, FP = 65, TN = 212, FN = 4) reflects improved case detection and overall classification fidelity. Variable-importance rankings (Fig. 4B) were broadly concordant with the full cohort, with amino-acid metabolites (glutamate, histidine, myo-inositol, lactate) and HDL-related measures remaining dominant contributors, alongside several fucosylated and bisected glycans (SI Table S9).

Odds-ratio estimates (Fig. 4C) recapitulated the effect patterns observed in the main analysis, with elevated amino-acid metabolites associated with increased odds of malignancy and reduced fucosylated glycans associated with lower odds-ratios. Correlation structures in non-cancer and cancer subgroups (Fig. 4D and E) were consistent with the full-cohort findings, demonstrating preserved modality-specific clustering and the characteristic inverse associations between glycan features and HDL measures in cancer. Collectively, these results indicate that exclusion of major comorbidities strengthens model performance while retaining the underlying metabolic and glycomic signature of malignancy.

### Preservation of cancer probability structure across heterogeneous comorbidities

To further characterise the relationship between cancer-associated metabolic signatures and overall non-specific disease burden, we examined joint diagnosis prediction regions for cancer and alternative non-cancer diagnoses within a shared molecular feature space. Cancer probabilities were derived from the SCAN cancer model trained on the cancer sub-cohort, while non-specific disease probabilities were generated using an independent classifier trained on alternative non-cancer diagnosis status, but constrained to the same metabolomic variables.

**Fig. 5:**
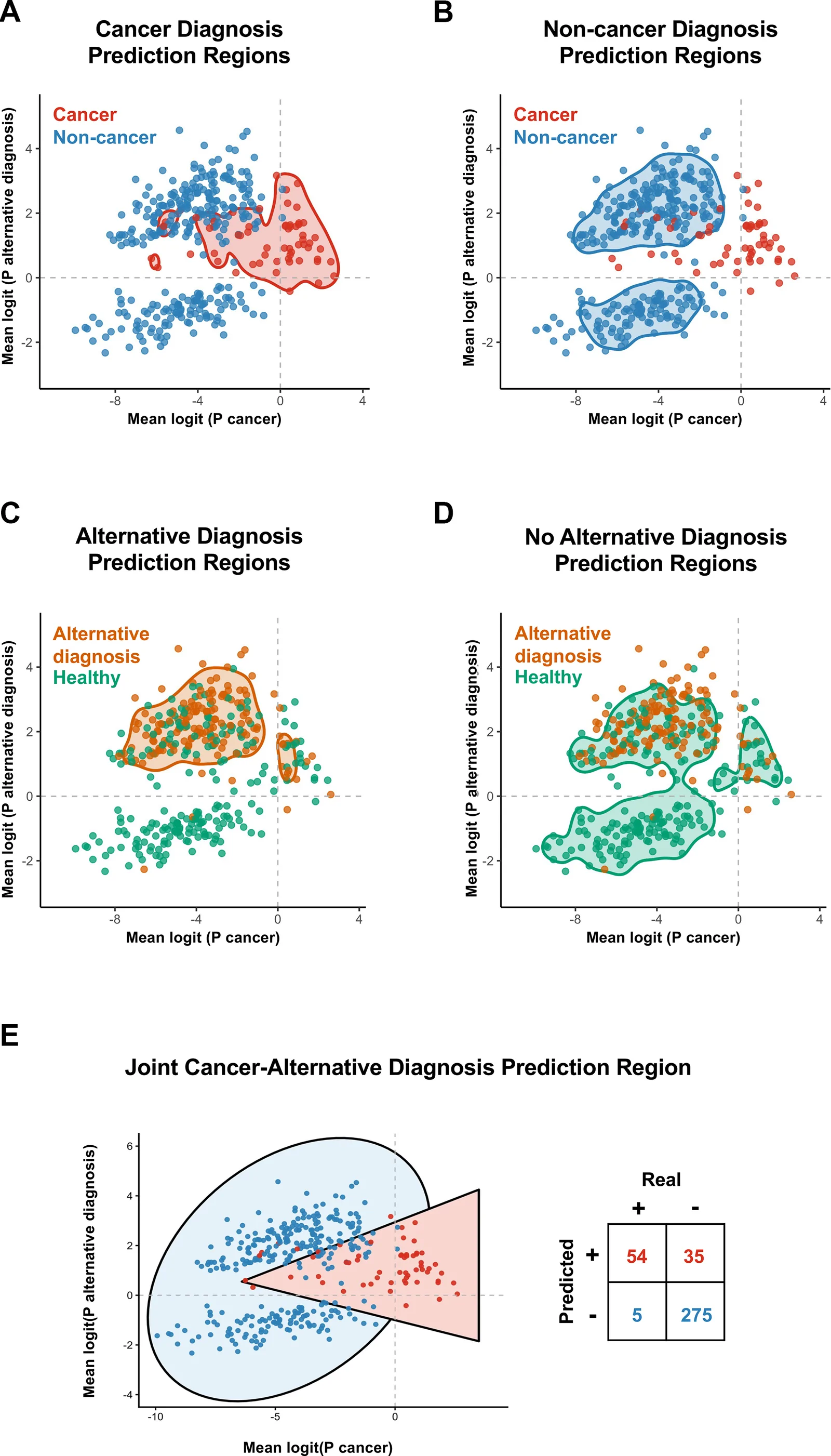
**Joint probability-space representation of cancer and alternative diagnosis predictions.** Participants are mapped into a joint logit-transformed probability space defined by predicted cancer risk and predicted alternative diagnosis risk. Predictions were generated by independent classifiers trained on a shared molecular feature set. **(A)** Cancer prediction regions, with participants coloured according to observed cancer status (cancer n = 59, non-cancer n = 310). **(B)** Cancer prediction regions shown using the same participants and probability space. **(C)** Alternative diagnosis prediction regions, with participants coloured according to observed alternative diagnosis status (alternative diagnosis n = 154, no alternative diagnosis n = 215). **(D)** Alternative diagnosis prediction regions shown using the same probability space, with participants coloured according to the absence of an alternative diagnosis. Kernel density contours enclose 75% of the probability mass. Dashed lines indicate zero on the logit scale. **(E)** Joint probability space showing the wedge-shaped decision region used for projection-based classification and the corresponding confusion matrix.

Projection of individuals into the joint logit-transformed probability space showed clear separation between cancer and non-cancer participants along the cancer probability axis (Fig. 5A and B), consistent with the strong discriminatory performance of the cancer model. Cancer cases clustered within a relatively compact region of the joint space, as reflected by a well-defined 75% kernel density contour. In contrast, predictions for alternative non-cancer diagnoses showed substantially greater dispersion and overlap (Fig. 5C and D), consistent with the biological and clinical heterogeneity of the non-malignant conditions represented in the cohort, including cardiovascular, renal, and respiratory disease. Despite this heterogeneity, the cancer-associated metabolic signal remained stable across the full spectrum of predicted comorbidity risk, indicating that cancer-related metabolic perturbations were preserved even in multimorbid individuals.

To enable downstream classification and quantitative assessment, an explicit geometric decision region was defined within the joint probability space. A directional wedge-shaped region derived from the cancer-associated kernel density distribution was constructed to capture the dominant axis of increasing cancer probability while accounting for concurrent alternative non-cancer diagnostic burden (Fig. 5E). Classification performance was broadly consistent across alternative wedge geometries (SI Fig. S14). Individuals within this region were classified as predicted cancer-positive, allowing construction of a confusion matrix and calculation of performance metrics. Under this projection-based decision rule, the model achieved high overall accuracy of 89.2% (95% CI 85.7–92.6), with balanced sensitivity and specificity of 91.5% (95% CI 87.1–93.5) and 88.7% (95% CI 84.3–93.1), respectively (Fig. 5E). These metrics reflect the internal consistency of cancer probability estimates within the joint space and illustrate how the continuous risk landscape can be mapped onto a discrete classification framework. Importantly, this analysis provides an interpretable representation of joint cancer-alternative non-cancer diagnosis risk rather than an independent validation of diagnostic performance; all probability estimates were derived from cross-validated predictions with strict separation of training and test data throughout.

### High-specificity discrimination of metastatic disease in a non-specific symptom cohort

**Fig. 6:**
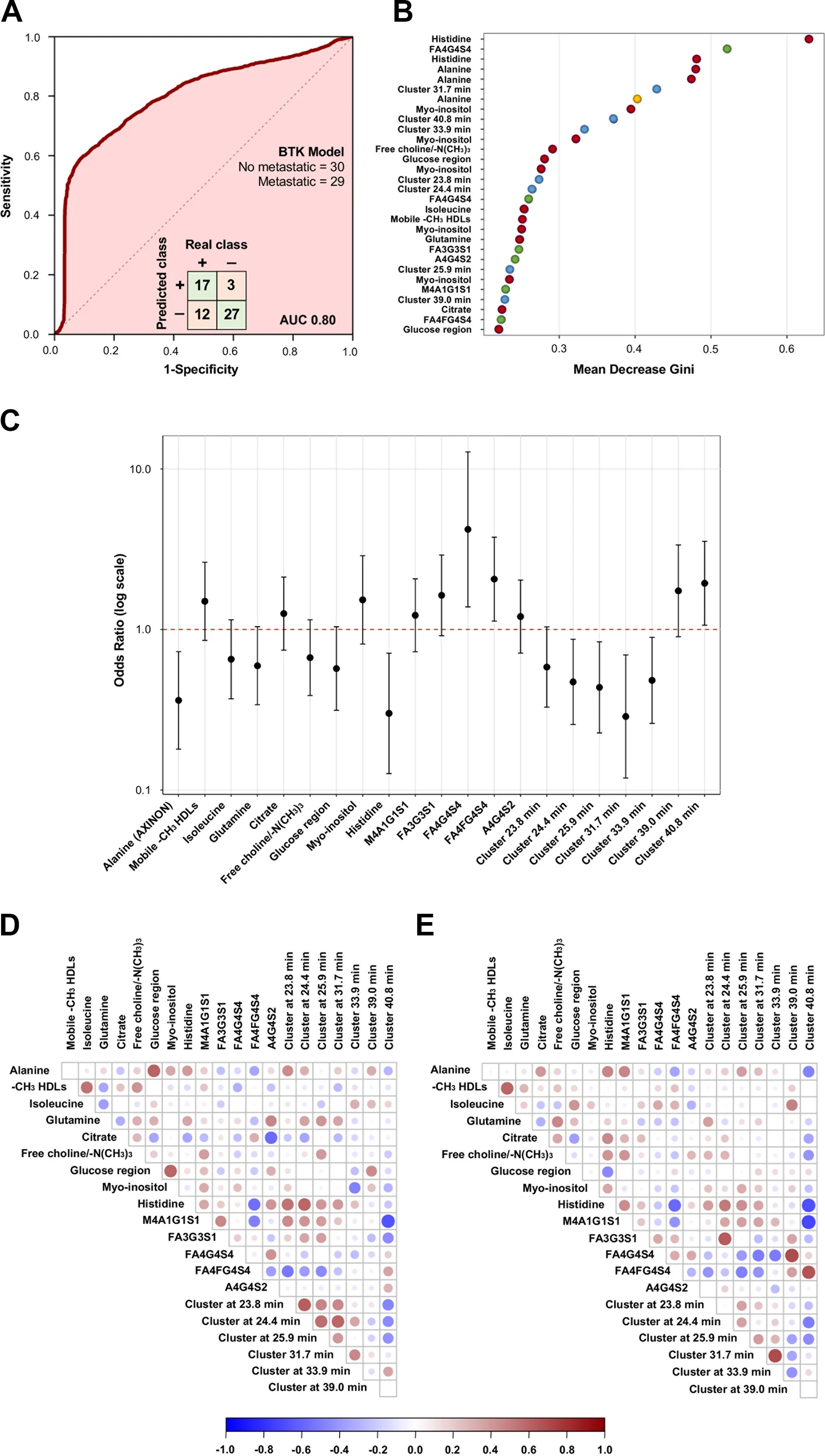
**Integrated metabolomic and glycomic analysis of metastatic and non-metastatic cancer. (A)** Receiver operating characteristic (ROC) curve for the integrated classification model combining untargeted NMR metabolomics, targeted *AXINON® System* metabolite quantification, and semi-targeted *N*-glycan profiling (n = 59; 29 metastatic, 30 non-metastatic). The inset shows the confusion matrix at the selected decision threshold. **(B)** Variable importance plot showing the top features ranked by mean decrease in Gini from the Random Forest classifier, including amino acids, lipoprotein-derived NMR measures, spectral regions, and glycan-associated features (SI Table S2). **(C)** Odds ratios (log scale) with 95% confidence intervals for the principal variables estimated using logistic regression. **(D)** Pearson correlation matrix of the selected metabolites and glycan features in non-metastatic cancer. **(E)** Pearson correlation matrix of the same variables in metastatic cancer. Positive correlations are shown in red and negative correlations in blue; circle size and colour intensity indicate the magnitude of the correlation coefficient.

Integrated metabolomic and glycomic profiling enabled discrimination between metastatic (n = 29) and non-metastatic (n = 30) cancer. The multivariable classification model achieved an AUC of 0.801 (95% CI 0.789–0.813), with an overall accuracy of 75.2% (95% CI 72.6–77.7) (Fig. 6A; Table 2). At a decision threshold of 0.575, the BTK model showed high specificity for non-metastatic disease (90.9% (95% CI 89.0–92.8)) and more moderate sensitivity for metastatic disease (59.4% (95% CI 57.6–61.2)). In absolute terms, this corresponded to 27 TN and 17 TP, with 3 FP and 12 FN. This performance profile reflects a clinically relevant trade-off in a non-specific symptom population, prioritising reliable exclusion of advanced malignancy while retaining meaningful sensitivity for metastatic disease.

Variable importance analysis from the integrated random forest model identified a heterogeneous panel of discriminatory features spanning amino acids, lipoprotein-derived NMR signals, spectral regions, and *N*-glycan measures (Fig. 6B; SI Table S10). The highest-ranked variables by mean decrease in Gini index included histidine, the complex tetra-antennary glycan FA4G4S4, alanine, and a fluorescence-derived glycan cluster eluting at 31.7 min, indicating contributions from both small-molecule metabolism and higher-order glycan structures. Additional features with stable multivariate importance comprised myo-inositol, mobile –CH_3_ HDL signals, glucose and free choline spectral regions, and multiple glycan-associated chromatographic clusters, consistent with the distributed nature of the metastatic signature.

**Fig. 7:**
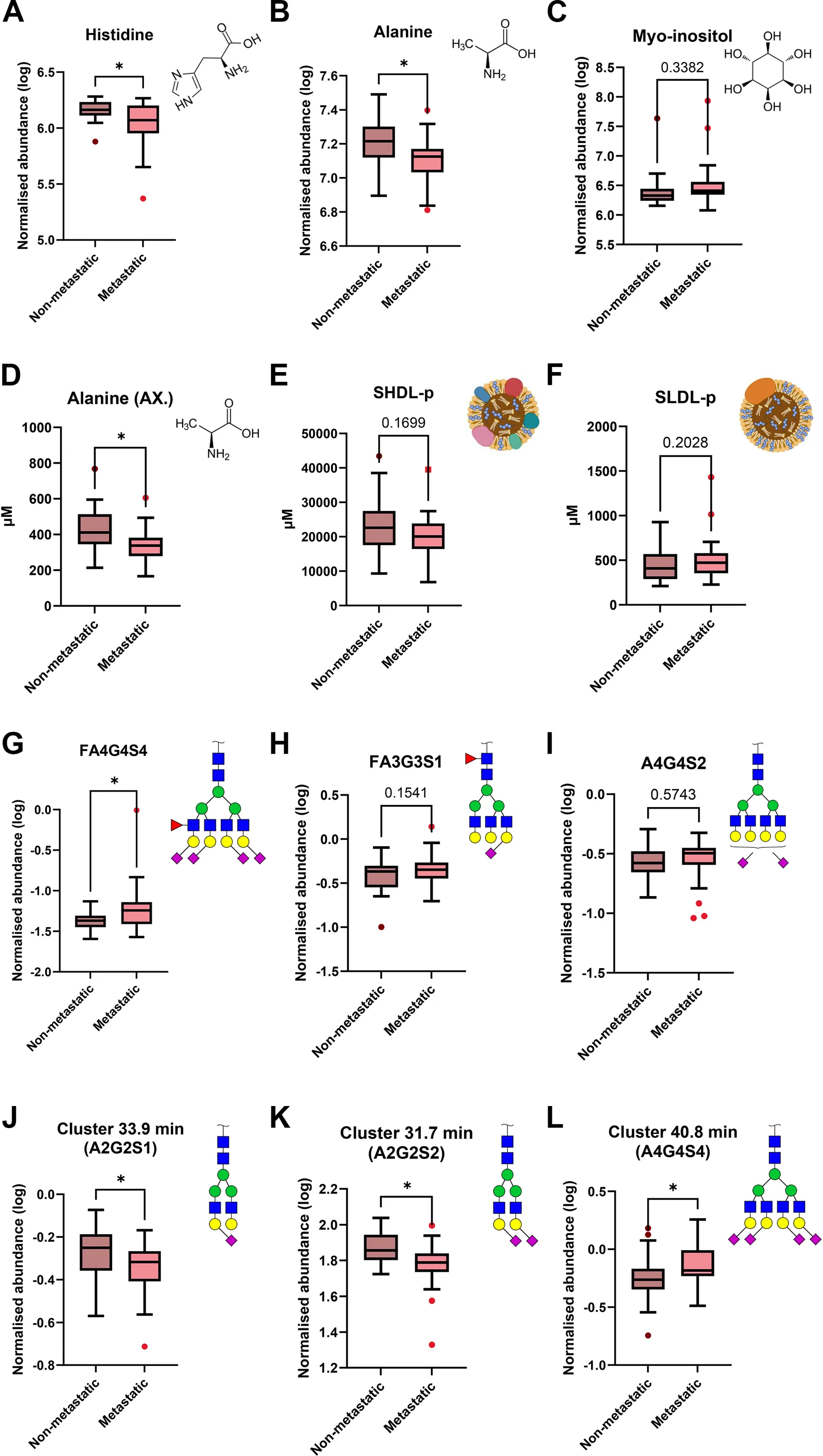
**Selected metabolomic, lipoprotein, and glycomic features in metastatic and non-metastatic cancer (n = 59; 29 metastatic, 30 non-metastatic).** Boxplots show the distribution of selected metabolites, lipoprotein measures, glycans, and glycan clusters in metastatic and non-metastatic cancer. **(A)** Histidine, **(B)** Alanine, **(C)** Myo-inositol, **(D)** Alanine quantified using the *AXINON® System* platform, **(E)** Small HDL particle concentration (SHDL-p), and **(F)** Small LDL particle concentration (SLDL-p) were selected from the integrated model based on Random Forest variable importance (mean decrease Gini). **(G)** FA4G4S4, **(H)** FA3G3S1, and **(I)** A4G4S2 were selected from the glycomics-only model integrating fluorescence and mass spectrometry data. **(J)** Cluster 33.9 min (predominantly A2G2S1), **(K)** Cluster 31.7 min (predominantly A2G2S2), and **(L)** Cluster 40.8 min (predominantly A4G4S4) correspond to fluorescence-derived glycan clusters. Statistical significance was assessed using unpaired two-sided *t*-tests with false discovery rate (FDR) correction for multiple testing. Asterisks indicate significance (p < 0.05; ∗p < 0.01).

Univariable analysis confirmed that several of the top-ranked features were individually associated with metastatic status (Figs. 6C and 7). Histidine showed a significant inverse association with metastatic disease (OR 0.41, 95% CI 0.21–0.81; p = 0.018; Fig. 7A), as did alanine measured by both untargeted NMR and targeted *AXINON® System* quantification (OR 0.36, 95% CI 0.18–0.73; p = 0.015; Fig. 7B–D). Myo-inositol, SHDL-p, and SLDL-p were also ranked among the most discriminatory features and showed weaker associations with metastatic cancer that did not survive multiple-testing correction (Fig. 7C–E, F). In contrast, selected *N*-glycan features were positively associated with metastatic status, including FA4G4S4 quantified by fluorescence detection (OR 2.33, 95% CI 1.25–4.33; p = 0.018) and by mass spectrometry (OR 4.20, 95% CI 1.38–12.76; p = 0.024). Fluorescence-derived glycan clusters eluting at 31.7 min and 40.8 min also remained significant following adjustment (p = 0.015 and 0.050, respectively). Although several additional metabolites, lipoprotein measures, and glycan clusters did not retain statistical significance after multiple-testing correction, their effect directions were concordant with multivariate importance rankings. Together, these findings indicate that discrimination between metastatic and non-metastatic disease arises from coordinated alterations across amino acid metabolism, lipoprotein-associated signals, and complex *N*-glycan architecture, supporting the value of integrative multi-omic modelling for stratifying disease burden.

Correlation analyses revealed marked differences in feature inter-relationships between non-metastatic and metastatic groups (Fig. 6D and E). Non-metastatic cases showed relatively structured correlation patterns, with moderate positive associations among amino acids and lipid-associated NMR signals (Spearman |r| ≈ 0.3–0.6). In contrast, metastatic disease was characterised by a reorganisation of correlation structure, including attenuation of metabolite–lipoprotein associations and strengthening of glycan–glycan correlations, several exceeding |r| ≈ 0.5, consistent with coordinated metabolic and glycomic reprogramming. Collectively, these coordinated alterations underpin the multivariate metastatic signature, yielding high specificity for non-metastatic disease (90.9%) and moderate sensitivity for metastatic disease (59.4%), supporting the utility of integrated metabolomic-glycomic profiling for disease stratification in non-specific symptom populations.

## Discussion

We demonstrate that malignancy is associated with a distinct and reproducible systemic molecular phenotype that can be distinguished from non-specific illness in patients presenting through real-world diagnostic pathways.^32^ Evidence for this phenotype was observed consistently across independent cohorts analysed using different platforms and modelling strategies. Such replication is notable given the limited clinical translation of many previously reported cancer metabolomic signatures, which has often been attributed to insufficient external validation, biological ambiguity, and analytical variability.^33^ Consistent with critiques of NMR-based cancer metabolomics, which emphasise that circulating metabolites reflect whole-organism physiology rather than tumour metabolism alone,^33^^,^^34^ our findings support a conserved host metabolic response to malignancy. A cancer-associated metabolic signature originally reported in the published SCAN1 cohort^14^ was independently reproduced in the larger and clinically more heterogeneous SCAN2 cohort, despite the use of a different analytical pipeline and pre-analytical controls. Conversely, metabolites independently prioritised in SCAN2 retained discriminatory performance and concordant effect directions when re-evaluated in SCAN1. Importantly, SCAN2 data were acquired on a commercial FDA-cleared 600 MHz *AXINON® System* NMR platform,^19^ arguing against cohort-, platform-, or procedure-specific effects. Together, these findings demonstrate that the metabolic phenotype was consistently identified across independent cohorts, analytical platforms, and complementary analytical approaches, supporting its robustness and generalisability. Notably, these findings should be considered in the context of existing liquid biopsy approaches for early cancer detection. Current strategies, including circulating tumour DNA (ctDNA) and protein-based assays, primarily detect tumour-derived signals, which may be limited in early-stage disease.^35^ In contrast, NMR-based metabolomic profiling captures a systemic host response to malignancy, integrating metabolic and inflammatory perturbations that are detectable even in heterogeneous, non-specific clinical presentations. This complementary perspective may be particularly valuable in non-site-specific diagnostic pathways.

SCAN2 integrates glycomic profiling alongside metabolomics to capture changes in protein glycosylation, a recognised hallmark of malignancy reflected in circulating acute-phase proteins, immunoglobulins, and hepatic glycoprotein.^36^^,^^37^ These changes are only indirectly captured by conventional NMR metabolomics, which primarily resolves small molecules and lipid subfractions. Incorporation of LC-MS-based glycan analysis therefore interrogates a complementary biochemical axis,^38^ yielding discriminatory features that are additive rather than redundant. Although the integrated model achieved only a modest, non-significant improvement in classification performance, the selection of glycan features indicates that glycomic and metabolomic data capture partially distinct aspects of the underlying biology.

Notably, exclusion of major metabolic confounders and selected malignancies improved classification performance while preserving the underlying biological signature. The top discriminatory variables were largely consistent between the full and refined models (SI Tables S8 and S9), with glycans (FA2G2S1, FA2BG1, M5A1G1S1), amino acids (glutamate, histidine), and HDL-related measures ranking prominently in both. This concordance indicates that improved performance reflects reduced masking of a conserved systemic metabolic and inflammatory signature associated with malignancy, rather than a distinct disease-specific profile. Comorbidities therefore introduce variability that attenuates, but does not alter, the cancer-associated phenotype.

Closer examination of the discriminatory features provides insight into the biological basis of model performance. Several complex biantennary *N*-glycans containing core fucosylation, terminal sialylation, or bisecting GlcNAc residues, including FA2G2S1, FA2G2S2, FA2BG1 and M5A1G1S1, were reduced in cancer. Altered fucosylation and sialylation are widely recognised features of malignancy; however, the direction of change is not uniform across glycan classes, carrier proteins, tumour types, or disease stages.^39^, ^40^, ^41^, ^42^ Both increases and decreases in specific fucosylated glycans have been reported in cancer, reflecting complex remodelling of circulating glycoproteins rather than a universal decrease or increase in fucosylation.^39^^,^^40^^,^^43^^,^^44^ The present findings therefore support cancer-associated alterations in systemic glycoprotein processing and immune-inflammatory signalling, consistent with broader host responses to malignancy.^42^^,^^45^

Detection of high-mannose and hybrid-type glycans (M5A1G1, M4A1G1S1) indicates disrupted glycan processing and increased protein turnover, features associated with systemic inflammation and malignancy.^46^, ^47^, ^48^, ^49^ In blood, these structures are typically interpreted as markers of dysregulated glycoprotein biosynthesis rather than direct tumour products, and their coexistence with fully sialylated glycans suggests a globally perturbed glycosylation landscape.^47^ These glycomic changes converge with NMR-derived markers of glycoprotein acetylation and lipoprotein remodelling.^50^^,^^51^ GlycA and GlycB index composite *N*-acetyl signals from circulating acute-phase proteins (α1-acid glycoprotein, haptoglobin, and α1-antitrypsin) and are linked to cancer and chronic inflammatory states,^52^ while concurrent alterations in HDL particle profiles are consistent with inflammation-driven changes in hepatic lipid handling.^51^ Notably, these changes in HDL levels are reflected in NMR-derived lipoprotein measures obtained using the *AXINON® System* platform. Although subclass definitions differ across platforms (e.g. Nightingale Health, Bruker B.I.-LISA), they capture comparable entities such as total HDL particle concentration and size-resolved subclasses, and the observed reductions are consistent with patterns reported across NMR-based lipoprotein profiling approaches.

The accompanying metabolic perturbations, including altered histidine, glutamate, and myo-inositol levels, were consistently observed across both SCAN1 and SCAN2, supporting their interpretation as components of a systemic host response to malignancy.^53^^,^^54^ Reduced circulating histidine has been widely reported in cancer and inflammatory states and is thought to reflect increased utilisation during immune activation and oxidative stress.^54^^,^^55^ Dysregulated glutamate is compatible with altered amino-acid flux and energy metabolism characteristic of cancer-associated metabolic reprogramming, while changes in myo-inositol have been linked to hepatic dysfunction, insulin signalling, and inflammation.^56^ Together, the coordinated disruption of glycomic, lipoprotein, and metabolic features points to convergent perturbation of inflammatory, hepatic, and metabolic pathways,^50^ providing a biologically plausible basis for the reproducibility of the integrated molecular signature across the cohorts examined.

A distinctive contribution of this study is the use of orthogonal biplot representations to separate molecular signals associated with general illness from those specific to malignancy. Rather than relying on a single cancer-vs.-all classifier, the modelling framework explicitly disentangles non-specific illness and cancer-associated perturbation by training independent axes before projecting all individuals into a shared joint space.^31^^,^^57^ This approach reflects the clinical reality of non-site-specific referral pathways, in which many patients are systemically unwell without malignancy, and avoids conflation of illness-related and cancer-specific biology.^6^ The resulting biplots define resolvable regions in which cancer cases cluster independently of the severity of generic illness, indicating that discrimination is driven by malignancy-associated metabolic and glycomic features rather than by overall systemic disturbance.^30^ Importantly, the observed classification performance arises not simply from model complexity, but from explicit structuring of biological variance into interpretable dimensions corresponding to illness and cancer.^30^ This representation enhances transparency and clinical plausibility by allowing individual patients to be contextualised across multiple biological processes rather than reduced to a single composite score.

The ability of the integrated approach to discriminate metastatic from non-metastatic disease represents an important secondary finding. Although sensitivity was lower than specificity, the strong rule-out performance is clinically relevant. As well as being highly reassuring for cancer survivors, patients presenting with non-specific symptoms often undergo extensive imaging to exclude disseminated disease.^3^ These findings suggest that blood-based molecular signatures may ultimately help identify patients unlikely to harbour metastatic disease, although this requires validation in larger cohorts.^6^^,^^7^ These observations are consistent with SCAN1 and support the interpretation that systemic metabolic disturbance scales with tumour burden.^14^

Several considerations temper these conclusions. Although this study represents a substantial expansion of the original SCAN cohort,^14^ the number of cancer cases–and particularly metastatic cases–remains modest, reflecting the real-world, non-enriched population of patients presenting with non-specific symptoms. As a result, validation in larger independent datasets will be required to confirm generalisability and refine model calibration.^29^ The dimensionality of the input feature space relative to the number of cancer cases can introduce a risk of overfitting, particularly in subgroup analyses such as the metastatic model. This was mitigated through fully nested cross-validation with feature selection performed within training folds and restriction to a reduced feature set. Consistent selection of key variables across models supports the stability of the underlying signal.

While pre-analytical handling was more rigorously standardised than in SCAN1, some real-world variability is unavoidable in fast-track diagnostic pathways; however, replication of the metabolic signature under these conditions supports biological robustness and clinical feasibility rather than undermining it. The integrated multi-omics model incorporates a relatively large number of features,^58^^,^^59^ and future work should assess whether a reduced panel of metabolites and glycans could retain comparable accuracy while facilitating clinical translation, particularly given the current requirement for specialised glycomic infrastructure.^60^ In addition, the glycan findings themselves require validation in larger, independent populations to confirm their generalisability and robustness. Finally, although patients with active cancer treatment or organ-specific diagnostic pathways were excluded, residual effects of comorbidity, medication use, or diet cannot be fully excluded.^61^ All participants underwent a minimum 2-h fast prior to blood collection; however, longer fasting periods may have further reduced biological variability. Recruitment occurred during the COVID-19 pandemic, and systematic SARS-CoV-2 status was not uniformly available. Patients were screened at the time of sampling, and those with a positive test would not have been admitted, making active infection unlikely. Additionally, information on medical treatments was not uniformly available, and residual treatment-related metabolic effects therefore cannot be excluded. Such influences are inherent to real-world cohorts and highlight the importance of prospective evaluation across diverse populations.

### Conclusion

This study demonstrates that integrated NMR metabolomics and LC-MS glycomics can discriminate cancer from non-cancer in patients presenting to primary care with non-specific symptoms. Replication of the SCAN1 metabolic signature in a larger, clinically heterogeneous cohort indicates a stable underlying biological signal across populations and sampling conditions. The addition of glycomic profiling provided complementary biological information, capturing cancer-associated alterations in glycosylation that are only weakly reflected in the metabolome and thereby extending insight into the systemic host response to malignancy. Combined with orthogonal biplot representations that separate cancer-specific biology from generic illness, this enhanced the biological interpretability of the integrated multi-omics approach in heterogeneous clinical settings. Together, these findings support blood-based multi-omics assessment as a feasible adjunct to existing diagnostic pathways, particularly as a triage tool for patients presenting with non-specific symptoms. By providing objective, quantifiable biomarkers alongside clinical judgement, this approach could help prioritise patients for imaging and specialist referral, reduce unnecessary investigations, and support earlier detection of malignancy in real-world primary care settings. Importantly, key components of this approach, including NMR-based lipoprotein profiling and glycomic assays, are already available in clinical or near-clinical settings, supporting the feasibility of translation into practice.

## Contributors

D. C. A., J. R. L., and S. A. conceived and designed the study. D. C. A., S. A., and the SCAN consortium were responsible for study oversight and patient recruitment. A. G. Y. and S. A. were involved in sample collection and processing. A. G. Y. and S. de J. performed NMR experiments. P. L. H. G. prepared the samples for glycomics analysis. J. C. and G. E.-H. performed the glycomics experiments and data processing. E. S. oversaw the NMR analysis. D. I. R. S. oversaw the glycomics analysis. T.K. and J. R. L. processed and analysed the data. T. K., J. J. M., and B. S. performed the multivariate analysis. T. K. drafted the manuscript. D. C. A. provided critical feedback and guidance for analysis design and manuscript writing. T. K. and D. C. A. accessed and verified the underlying data. All authors reviewed and approved the final manuscript.

## Data sharing statement

The study protocol, anonymised individual-level processed data, data dictionary, and analytical code are available from the corresponding authors upon reasonable request. Individual-level data will be shared in a de-identified form. The raw data include proprietary NMR metabolomics datasets generated by numares AG and cannot be made publicly available. Access to these data may be provided to qualified researchers upon reasonable request, subject to approval by numares AG and, where required, a confidentiality agreement.

## Declaration of interests

J. C. is an employee of Ludger Ltd, UK; G. E.-H. is an employee of Ludger Ltd, UK; D. I. R. S. is an employee of Ludger Ltd, UK. S. d. J. was an employee of numares, AG. E. S. is an employee of numares, AG. J. R. L. previously held shares in Oxomics Ltd, a University of Oxford spinout company that is no longer trading.

## Appendix A Supplementary data

SCAN Omics SI

SI Dataset1 Targeted NMR metabolomics data generated using the *AXINON® System* for all participants in the SCAN2 cohort.
