# Supplementary material for: NMR metabolomics and glycomics for cancer detection in patients with non-specific symptoms: a prospective observational cohort study
Source: eBioMedicine. 2026 Aug 14;131:106434. doi: 10.1016/j.ebiom.2026.106434 (PMC13503105; doi:10.1016/j.ebiom.2026.106434)
Supplement: Supplementary file 1 [file mmc1.pdf]

Supplementary Information

NMR metabolomics and glycomics for cancer detection in patients with non-specific symptoms: a prospective observational cohort study

Tereza Kacerova[1,2,3*,†], Abi G. Yates[4†], James R. Larkin[4], Boris Shulgin[4], Jack J. J. J. Miller[5,6], Philippa L. Harris Gleave[4], Sebastian de Jel[7], Jack Cheeseman[8], Georgia Elgood-Hunt[8], the SCAN Consortium[9#], Eric Schiffer[7], Daniel I. R. Spencer[8], Suzie Anthony[10], Daniel C. Anthony[4*]

[1] *Chemistry Research Laboratory, Department of Chemistry, University of Oxford, Oxford OX1 3TA, UK*
[2] *Kavli Institute of Nanoscience Discovery, Dorothy Crowfoot Hodgkin Building, Oxford OX1 3QU, UK*
[3] *Physical and Theoretical Chemistry, University of Oxford, Oxford OX1 3QZ, UK*
[4] *Department of Pharmacology, University of Oxford, Oxford OX1 3QT, UK*
[5] *Oxford Centre for Magnetic Resonance Research, University of Oxford, Oxford OX3 9DU, UK*
[6] *Department of Clinical Medicine, Aarhus University, Aarhus 8200, Denmark*
[7] *numares AG, Regensburg 93053, Germany*
[8] *Ludger Ltd, Culham Science Centre, Abingdon OX14 3EB, UK*
[9] *SCAN Consortium, Oxford University Hospitals NHS Foundation Trust and University of Oxford, Oxford, UK*
[10] *Interventional Oncology, Department of Radiology, University of Oxford, Oxford OX3 9DU, UK*

* *Corresponding authors*

Daniel C. Anthony (clinical and biological)

Department of Pharmacology, University of Oxford, Oxford OX1 3QT, UK
daniel.anthony@pharm.ox.ac.uk
Tel: +44(0)1865 281135

Tereza Kacerova (analytical chemistry and data analysis)

Chemistry Research Laboratory, Department of Chemistry, University of Oxford, Oxford OX1 3TA, UK
tereza.kacerova@chem.ox.ac.uk
Tel: +44(0)7938133474

†These authors contributed equally

\# Members listed at the end of the Article

Detected NMR Metabolites and Lipoproteins

SI Table 1 Putative assignments of untargeted CPMG-derived NMR spectral features. Features were obtained from CPMG spectra used for binning and downstream modelling, enabling improved resolution of small-molecule metabolites through suppression of macromolecular signals. Assignments were supported by prior experimental data, literature, and the Human Metabolome Database. Overlapping signals are reported as composite features.

Feature class	Assignment
Lipoprotein / lipid-associated signals	mobile $-CH_3$ HDL/LDL (broad)
Lipoprotein / lipid-associated signals	mobile $-CH_3$ Chyl/VLDL (broad)
Lipoprotein / lipid-associated signals	mobile $(-CH_2-)n$ LDL (broad)
Lipoprotein / lipid-associated signals	mobile $(-CH_2-)n$ Chyl/VLDL (broad)
Lipoprotein / lipid-associated signals	mobile $(-CH_2-)n$ lipids (broad)
Lipoprotein / lipid-associated signals	mobile $-N(CH_3)_3$ lipoprotein signal (singlet)
Lipoprotein / lipid-associated signals	unsaturated lipid (multiplet)
Lipoprotein / lipid-associated signals	lipoprotein (broad)
Glycoprotein	NAC1 (singlet)
Glycoprotein	NAC2 (singlet)
Amino acids	isoleucine (multiplet)
Amino acids	leucine (multiplet)
Amino acids	valine (doublet)
Amino acids	branched-chain amino acids (BCAAs; multiplet)
Amino acids	alanine (doublet)
Amino acids	lysine (multiplet)
Amino acids	arginine (multiplet)
Amino acids	proline (multiplet)
Amino acids	glutamate (multiplet)
Amino acids	glutamine (multiplet)
Amino acids	glutamine/glutamate (overlapping; multiplet)
Amino acids	histidine (multiplet)
Amino acids	threonine (doublet)
Amino acids	phenylalanine (multiplet)
Amino acids	tyrosine (multiplet)
Organic acids / ketone bodies	lactate (doublet)
Organic acids / ketone bodies	citrate (multiplet)

Organic acids / ketone bodies	acetate (singlet)
Organic acids / ketone bodies	acetoacetate (singlet)
Organic acids / ketone bodies	3-hydroxybutyrate (multiplet)
Other metabolites	creatine (singlet)
Other metabolites	creatinine (singlet)
Other metabolites	free choline (singlet)
Other metabolites	myo-inositol (multiplet)
Other metabolites	scyllo-inositol (singlet)
Other metabolites	α-, β-glucose (multiplet)
Other metabolites	urea (singlet)
Other metabolites	formate (singlet)
Composite / overlapping regions	lactate and mobile lipid signal
Composite / overlapping regions	valine/proline
Composite / overlapping regions	lysine/acetate
Composite / overlapping regions	arginine/lysine/leucine
Composite / overlapping regions	lysine/creatinine/creatine
Composite / overlapping regions	mobile -$N(CH_3)_3$/free choline
Composite / overlapping regions	mobile -$N(CH_3)_3$/ β-glucose
Composite / overlapping regions	mobile -$N(CH_3)_3$/ β-glucose/myo-inositol
Composite / overlapping regions	α-glucose/unsaturated lipid
Composite / overlapping regions	glutamine/glutamate/unsaturated lipid region
Composite / overlapping regions	NAC1/lipid-associated methylene region

SI Table 2 Explanation of glycan nomenclature used throughout the manuscript. Glycan structures are reported using the Oxford glycan nomenclature. F denotes core fucosylation, A the number of antennae (branches), G the number of galactose residues, S the number of sialic acid residues, B a bisecting N-acetylglucosamine residue, and M the number of mannose residues. Representative examples of the principal glycans discussed in the manuscript are provided.

**Symbol**	**Meaning**
F	Core fucose
A	Number of antennae (branches)
G	Number of galactose residues
S	Number of sialic acid residues
B	Bisecting N-acetylglucosamine residue
M	Mannose residue
**Glycan**	**Description**
FA1G1S1	Core-fucosylated monoantennary glycan with one galactose and one sialic acid
FA2G2	Core-fucosylated biantennary digalactosylated glycan
FA2G2S1	Core-fucosylated biantennary digalactosylated monosialylated glycan
FA2G2S2	Core-fucosylated biantennary digalactosylated disialylated glycan
FA2BG1	Core-fucosylated biantennary glycan containing a bisecting N-acetylglucosamine and one galactose residue
FA2BG1S1	Core-fucosylated biantennary glycan containing a bisecting N-acetylglucosamine, one galactose and one sialic acid
FA2BG2	Core-fucosylated biantennary glycan containing a bisecting N-acetylglucosamine and two galactose residues
M4A1G1S1	Hybrid glycan containing four mannose residues, one antenna, one galactose residue and one sialic acid
M5A1G1	Hybrid glycan containing five mannose residues, one antenna and one galactose residue
M5A1G1S1	Hybrid glycan containing five mannose residues, one antenna, one galactose residue and one sialic acid
FA3G3S1	Core-fucosylated triantennary glycan with three galactose residues and one sialic acid
FA3G3S3	Core-fucosylated triantennary glycan with three galactose residues and three sialic acids
A2G2S2	Biantennary digalactosylated disialylated glycan
A3G3S2	Triantennary glycan with three galactose residues and two sialic acids
A3G3S3	Triantennary glycan with three galactose residues and three sialic acids
A4G4S4	Tetra-antennary glycan with four galactose residues and four sialic acids
FA4G4S4	Core-fucosylated tetra-antennary glycan with four galactose residues and four sialic acids
FA4FG4S4	Multi-fucosylated tetra-antennary glycan with four galactose residues and four sialic acids

SI Table 3 List of all AXINON-derived NMR parameters included in downstream analyses. Parameters were obtained from the *AXINON® lipoFIT®* platform and associated *AXINON® nephroPanel* analyses derived from the same underlying NMR acquisition. The table includes lipoprotein-related parameters, amino acids, renal markers, and additional low-molecular-weight metabolites used for multivariate modelling.

AXINON Parameter	Source Panel	Description
lipoFIT-S100_RUO_Alanine	AXINON® lipoFIT®	Alanine concentration
lipoFIT-S100_RUO_Valine	AXINON® lipoFIT®	Valine concentration (branched-chain amino acid, BCAA)
lipoFIT-S100_RUO_Leucine	AXINON® lipoFIT®	Leucine concentration (BCAA)
lipoFIT-S100_RUO_Isoleucine	AXINON® lipoFIT®	Isoleucine concentration (BCAA)
lipoFIT-S100_RUO_LDL-p	AXINON® lipoFIT®	Total LDL particle concentration
lipoFIT-S100_RUO_HDL-p	AXINON® lipoFIT®	Total HDL particle concentration
lipoFIT-S100_RUO_SLDL-p	AXINON® lipoFIT®	Small LDL particle concentration
lipoFIT-S100_RUO_LHDL-p	AXINON® lipoFIT®	Large HDL particle concentration
lipoFIT-S100_RUO_LVLDL-p	AXINON® lipoFIT®	Large VLDL particle concentration
lipoFIT-S100_RUO_LLDL-p	AXINON® lipoFIT®	Large LDL particle concentration
lipoFIT-S100_RUO_SHDL-p	AXINON® lipoFIT®	Small HDL particle concentration
lipoFIT-S100_RUO_VLDL-s	AXINON® lipoFIT®	Mean VLDL particle size (diameter)
lipoFIT-S100_RUO_LDL-s	AXINON® lipoFIT®	Mean LDL particle size (diameter)
lipoFIT-S100_RUO_HDL-s	AXINON® lipoFIT®	Mean HDL particle size (diameter)
lipoFIT-S100_RUO_Triglycerides	AXINON® lipoFIT®	Total triglyceride concentration
lipoFIT-S100_RUO_HDL-Cholesterol	AXINON® lipoFIT®	HDL cholesterol
lipoFIT-S100_RUO_LDL-Cholesterol	AXINON® lipoFIT®	LDL cholesterol
lipoFIT-S100_RUO_Total-Cholesterol	AXINON® lipoFIT®	Total cholesterol
lipoFIT-S100_RUO_VLDL-c	AXINON® lipoFIT®	Cholesterol carried in VLDL particles
lipoFIT-S100_RUO_IDL-c	AXINON® lipoFIT®	Cholesterol carried in IDL particles
lipoFIT-S100_RUO_LDL-c	AXINON® lipoFIT®	Cholesterol carried in all LDL subclasses combined
lipoFIT-S100_RUO_LDL.A-c	AXINON® lipoFIT®	Cholesterol in LDL subclass A (largest LDL particles)
lipoFIT-S100_RUO_LDL.B-c	AXINON® lipoFIT®	Cholesterol in LDL subclass B (intermediate LDL particles)

lipoFIT-S100_RUO_LDL.C-c	AXINON® lipoFIT®	Cholesterol in LDL subclass C (smallest/densest LDL particles)
lipoFIT-S100_RUO_HDL.A-c	AXINON® lipoFIT®	Cholesterol in HDL subclass A (largest HDL particles)
lipoFIT-S100_RUO_HDL.B-c	AXINON® lipoFIT®	Cholesterol in HDL subclass B (intermediate HDL particles)
lipoFIT-S100_RUO_HDL.C-c	AXINON® lipoFIT®	Cholesterol in HDL subclass C (smallest HDL particles)
AXINON_nephroPanel_Plus_Creatinine	AXINON® nephroPanel	Creatinine concentration
AXINON_nephroPanel_Plus_Valine	AXINON® nephroPanel	Valine concentration
AXINON_nephroPanel_Plus_Isoleucine	AXINON® nephroPanel	Isoleucine concentration
AXINON_nephroPanel_Plus_Glycerol	AXINON® nephroPanel	Glycerol concentration
AXINON_nephroPanel_Plus_Creatine	AXINON® nephroPanel	Creatine concentration
AXINON_nephroPanel_Plus_Dimethylamine	AXINON® nephroPanel	Dimethylamine concentration
AXINON_nephroPanel_Plus_Dimethylsulfone	AXINON® nephroPanel	Dimethylsulfone concentration
AXINON_nephroPanel_Plus_Myo-Inositol	AXINON® nephroPanel	Myo-inositol concentration

**Suffix**	**Meaning**
-p	Particle concentration (particle number)
-s	Mean particle size
-c	Cholesterol concentration/content within the retrospective lipoprotein subclass

SI Table 4 Mean concentrations and standard deviations of *AXINON® lipoFIT®* and *AXINON® nephroPanel* parameters in non-cancer and cancer groups. Values are presented as average concentrations (µM) and corresponding standard deviations for amino acids, lipoprotein subclasses, cholesterol fractions, and triglyceride-related parameters measured by NMR spectroscopy.

**AXINON parameter**	**Non-cancer**		**Cancer**		**t-test**
	**Mean (µM)**	**SD (µM)**	**Mean (µM)**	**SD (µM)**	**p-value**
lipoFIT-S100_RUO_Alanine	423	108	385	111	0.0136
lipoFIT-S100_RUO_Valine	238	52	214	48	0.0014
lipoFIT-S100_RUO_Leucine	134	31	125	25	0.0507
lipoFIT-S100_RUO_Isoleucine	82	17	81	15	0.8177
lipoFIT-S100_RUO_LDL-p	1004	359	953	397	0.3202
lipoFIT-S100_RUO_HDL-p	34598	6807	28221	8213	<0.0001
lipoFIT-S100_RUO_SLDL-p	474	199	483	214	0.7648
lipoFIT-S100_RUO_LHDL-p	8241	3617	8002	3369	0.6395
lipoFIT-S100_RUO_LVLDL-p	5	3	4	4	0.3717
lipoFIT-S100_RUO_LLDL-p	626	193	597	175	0.2752
lipoFIT-S100_RUO_SHDL-p	27588	6804	21518	7158	<0.0001
lipoFIT-S100_RUO_VLDL-s	50	3	50	4	0.1132
lipoFIT-S100_RUO_LDL-s	21	0	21	0	0.8174
lipoFIT-S100_RUO_HDL-s	9	1	9	1	0.3125
lipoFIT-S100_RUO_Triglycerides	127	59	114	55	0.1181
lipoFIT-S100_RUO_HDL-Cholesterol	58	14	52	16	0.0047
lipoFIT-S100_RUO_LDL-Cholesterol	91	30	85	37	0.2047
lipoFIT-S100_RUO_Total-Cholesterol	170	40	157	47	0.0261
lipoFIT-S100_RUO_VLDL-c	22	8	21	7	0.2382
lipoFIT-S100_RUO_IDL-c	40	11	37	13	0.0230
lipoFIT-S100_RUO_LDL-c	99	23	94	26	0.1193
lipoFIT-S100_RUO_LDL.A-c	34	8	32	10	0.1012
lipoFIT-S100_RUO_LDL.B-c	19	7	18	7	0.2780
lipoFIT-S100_RUO_LDL.C-c	4	2	4	2	0.2146
lipoFIT-S100_RUO_HDL.A-c	18	6	18	6	0.9235
lipoFIT-S100_RUO_HDL.B-c	17	3	17	2	0.8169
lipoFIT-S100_RUO_HDL.C-c	21	7	16	8	<0.0001
AXINON_nephroPanel_Plus_Creatinine	92	54	100	32	0.2711
AXINON_nephroPanel_Plus_Valine	246	52	223	46	0.0023
AXINON_nephroPanel_Plus_Isoleucine	75	18	77	15	0.5271
AXINON_nephroPanel_Plus_Glycerol	179	68	209	114	0.0051
AXINON_nephroPanel_Plus_Creatine	28	17	31	24	0.2920
AXINON_nephroPanel_Plus_Dimethylamine	4	2	5	2	0.0230
AXINON_nephroPanel_Plus_Dimethylsulfone	15	10	17	11	0.1883
AXINON_nephroPanel_Plus_Myo-Inositol	64	22	73	28	0.0057

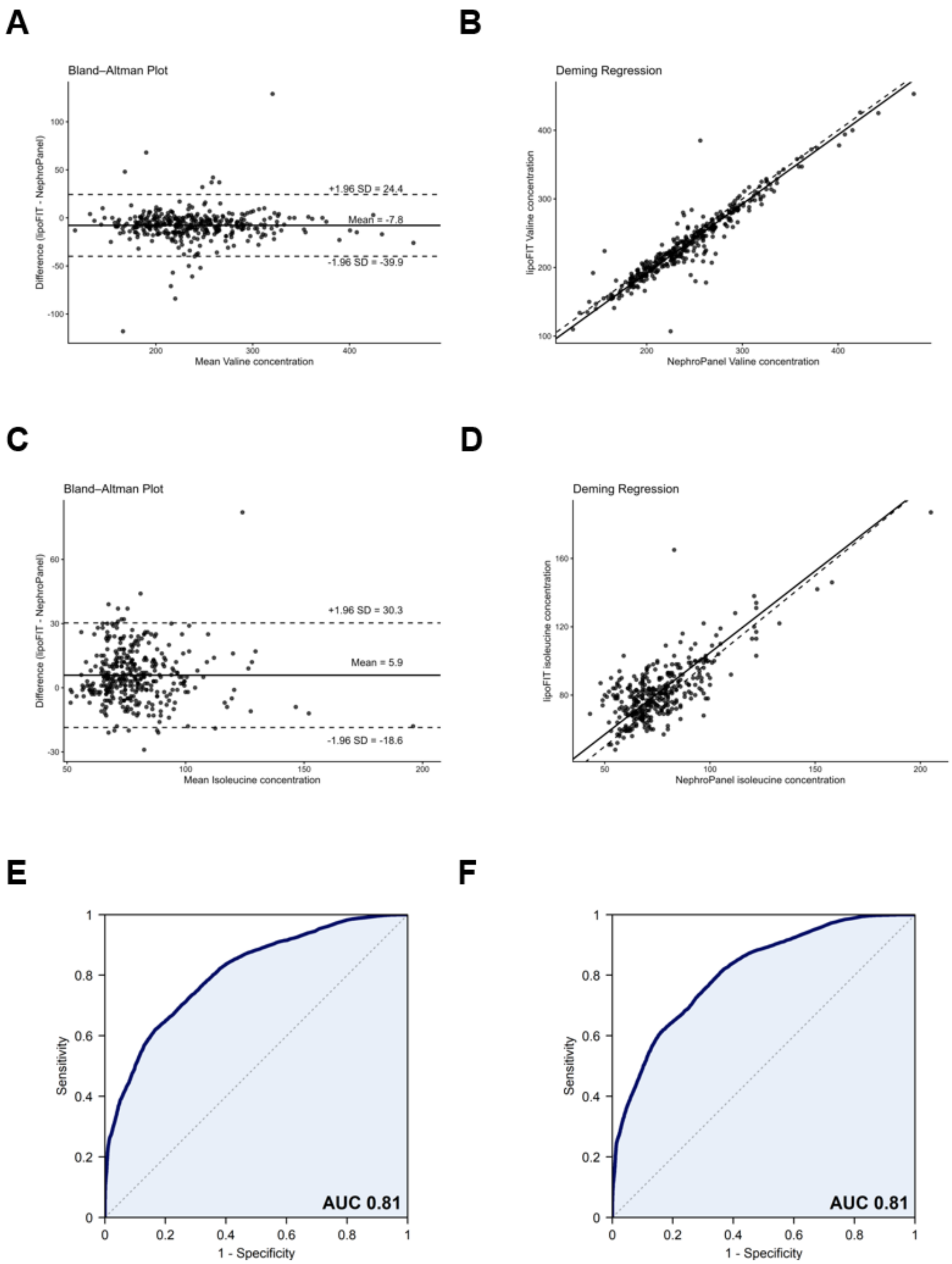

SI Figure 1 Agreement between valine and isoleucine concentrations quantified using the *AXINON® lipoFIT®* and *AXINON® nephroPanel* platforms. Bland-Altman plots comparing valine **(A)** and isoleucine **(C)** concentrations measured by the two independent quantification pipelines. Solid lines indicate the mean bias and dashed lines indicate the 95% limits of agreement. Deming regression analyses for valine **(B)** and isoleucine **(D)** demonstrate strong agreement between the two implementations, with slopes close to unity and only small constant offsets. To assess whether inclusion of both measurements influenced model performance, machine-learning analyses were repeated using either the *lipoFIT®* measurements alone or the *nephroPanel* measurements alone. The resulting diagnostic performance was essentially unchanged, with ROC AUCs of 0.810 (95% CI 0.803–0.815) for *lipoFIT®*-derived measurements **(E)** and 0.812 (95% CI 0.806–0.818) for *nephroPanel*-derived measurements **(F)**, indicating that predictive performance was not driven by duplicate measurements of valine and isoleucine. ROC, receiver-operating characteristic; AUC, area under the curve. r, Pearson correlation coefficient.

BTK dual-outcome prediction framework

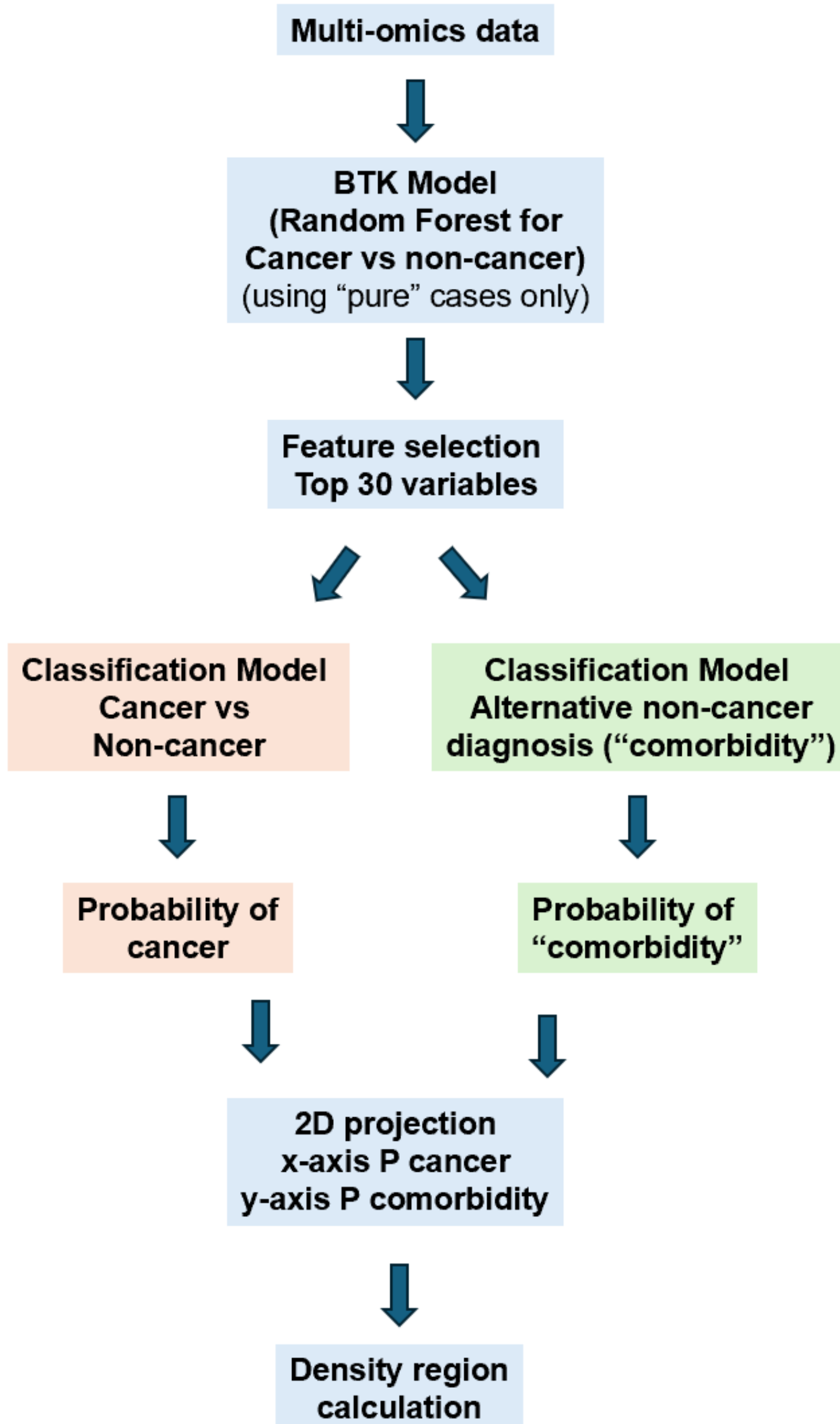

SI Figure 2 Schematic overview of the dual-outcome BTK modelling framework. Multi-omics data (NMR metabolomics and glycomics) were first subjected to feature selection within a cancer-focused subset to define a shared reduced feature space (top 30 variables). Two independent random forest models were then trained using this feature set: one to classify cancer vs non-cancer and one to distinguish alternative non-cancer diagnoses from healthy controls. Predicted probabilities from both models were generated using cross-validation and combined for each individual. These were subsequently represented in a two-dimensional space defined by the probability of cancer and the probability of alternative non-cancer diagnosis, enabling joint interpretation of disease-related molecular signals.

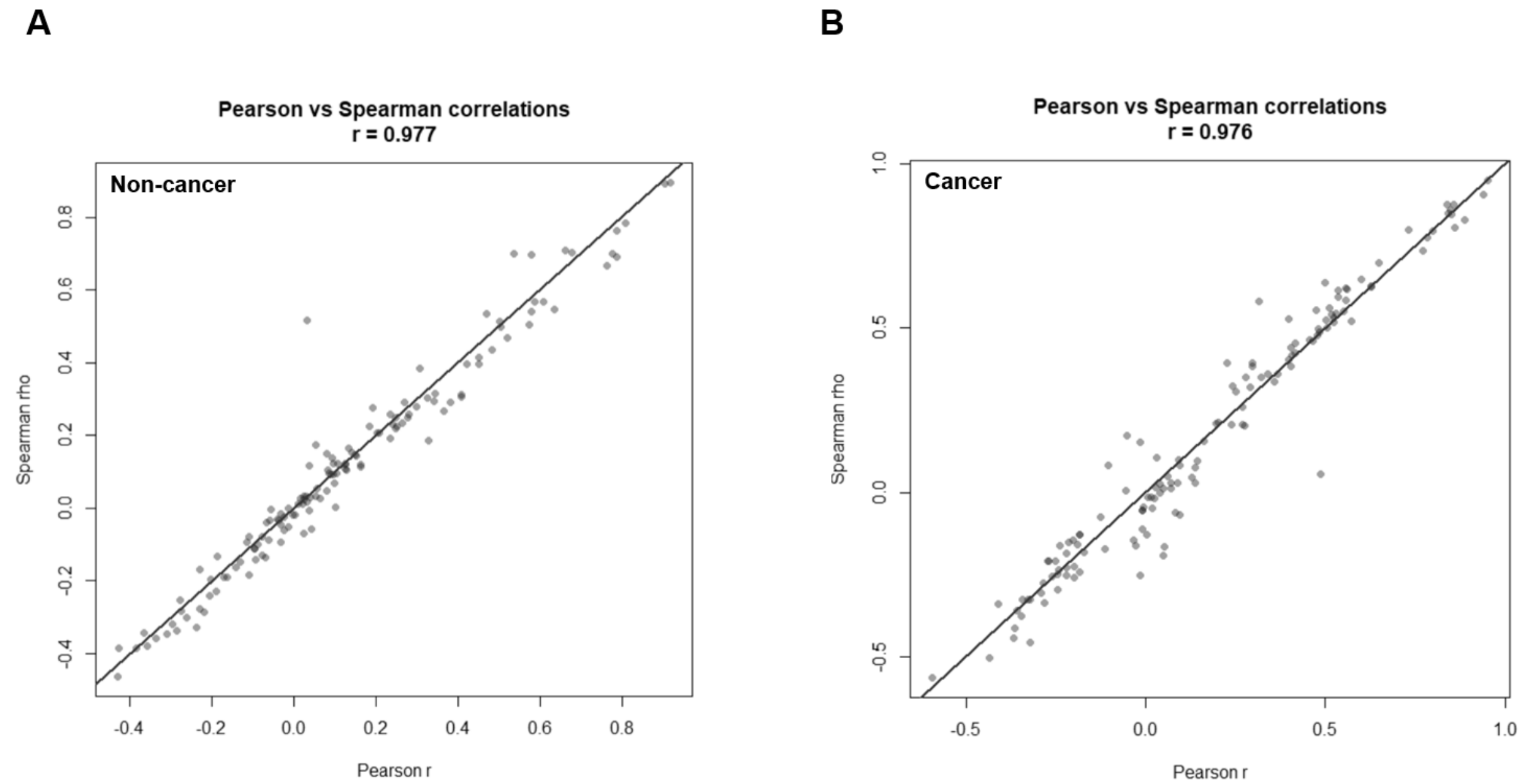

SI Figure 3 Comparison of Pearson and Spearman correlation coefficients. Pairwise correlations between variables included in the non-cancer (n = 310; **A**) and cancer (n = 59; **B**) correlation network analyses calculated using Pearson and Spearman correlation coefficients. Each point represents a single pairwise feature correlation and the red line indicates the line of identity (y = x). Pearson and Spearman correlation matrices showed strong agreement for both the non-cancer network (r = 0.977; mean absolute difference = 0.040) and the cancer network (r = 0.976; mean absolute difference = 0.055). At the correlation threshold used for network construction (|r| > 0.5), 17 of 19 edges were shared between the Pearson- and Spearman-based non-cancer networks (89.5% overlap), while all Pearson-derived edges were retained in the Spearman-based cancer network (32/32 shared edges; 100% overlap). These results indicate that the overall network structure was largely unaffected by the choice of correlation metric.

Demographics

SI Table 5 Distribution of cancer diagnoses in the SCAN2 cohort. Cancer types are shown with corresponding metastatic status at the time of assessment and participant counts. The cohort comprises a broad range of solid and haematological malignancies, reflecting the heterogeneity of a non-site-specific cancer detection setting. Metastatic status was defined according to clinical staging and imaging data available at recruitment.

Cancer	Metastatic	Count
Adenocarcinoma (not specified)	yes	4
Adenocarcinoma (colon)	yes	2
Adenocarcinoma (ovarian)	yes	1
Adenocarcinoma (lung)	no	1
Adenocarcinoma (pancreas)	yes	1
Adenocarcinoma (pancreas)	no	2
Adenocarcinoma (prostate)	yes	2
Adenocarcinoma (prostate)	no	3
B cell lymphoma	no	1
B cell lymphoma	yes	6
Basal cell carcinoma	no	2
Bladder cancer	no	1
Breast carcinoma	no	1
Carcinoid	no	1
Cervical cancer	yes	1
Cholangiocarcinoma	yes	2
Clear cell carcinoma kidney	yes	1
Ductal carcinoma in situ	no	1
Endometroid endometrial carcinoma grade 1	no	1
Epithelioid mesothelioma	yes	1
Hepatocellular carcinoma	no	1
Gastrointestinal stromal tumour	no	1
Lung cancer	yes	1
Lung cancer	no	1
Mesothelioma	no	1
Myelodysplasia	no	1
Myeloma	no	1
Neuroendocrine	no	1
Pancreatic	yes	1
Papillary transitional cell carcinoma	yes	1
Prostate cancer	no	6
Prostate cancer	yes	1
Renal cell carcinoma	no	1
Renal cell carcinoma	yes	3
Squamous cell carcinoma	no	1
Unknown primary cancer	yes	1
Unknown cancer	no	1

Assessment of sex as a potential confounder

To assess the potential influence of sex, the primary classification model was re-run with sex included as a covariate. Model performance remained unchanged (AUC 0.812, 95% CI 0.806–0.819; accuracy 68.8% (95% CI: 67.8-69.8)), indicating that the discriminatory signal is not driven by sex.

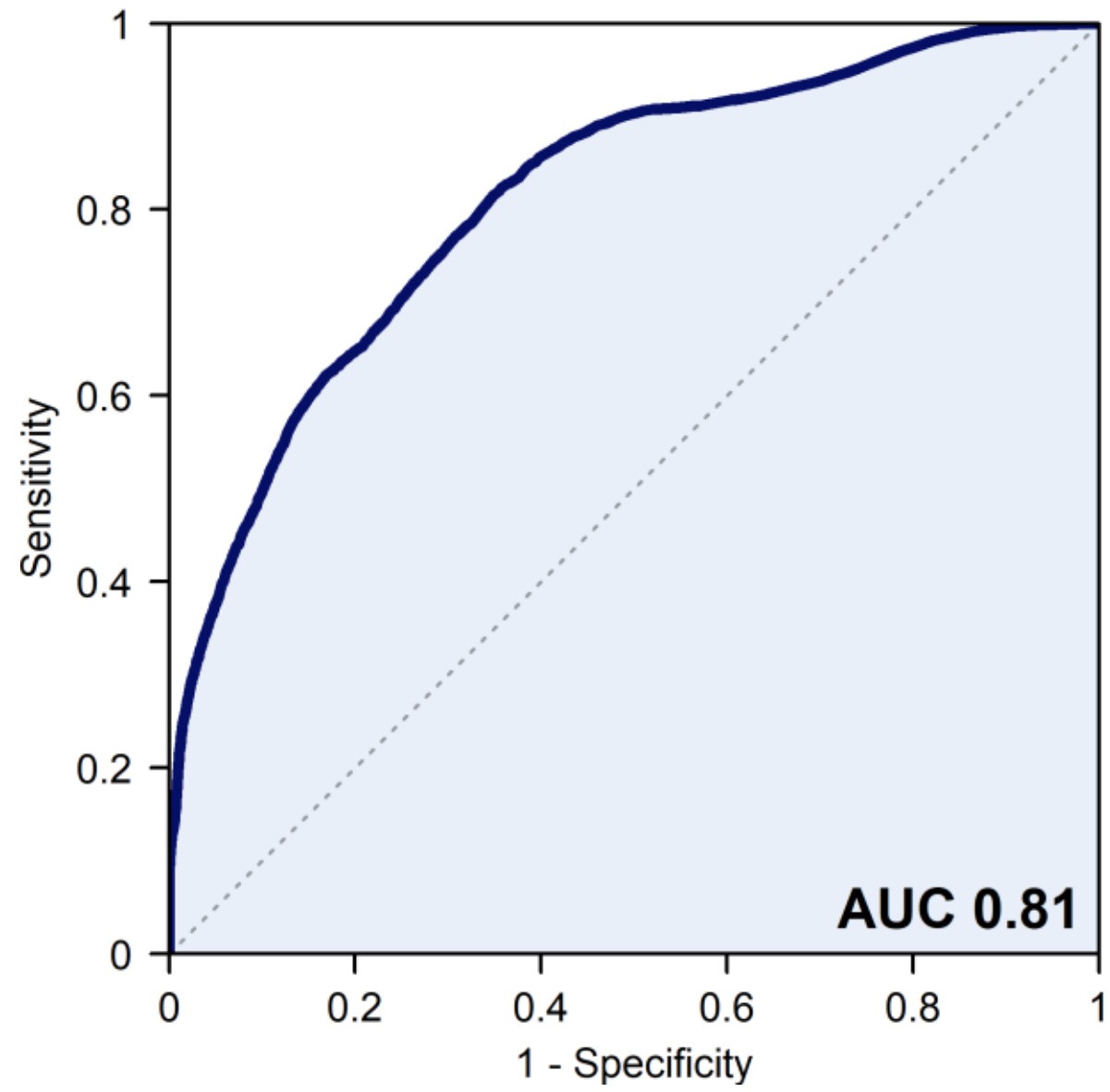

SI Figure 4 ROC curve for the primary model including sex as a covariate. Inclusion of sex did not materially affect model performance (AUC 0.814), supporting that the observed classification is not confounded by sex. ROC, receiver-operating characteristic; AUC, area under the curve.

Assessment of sex as a potential confounder

To assess potential sex-specific effects, the primary model was re-run separately in males and females. Model performance remained strong in both groups, with an AUC of 0.818 (95% CI 0.811–0.826) in males and 0.840 (95% CI 0.831–0.848) in females, supporting that the identified molecular signature is consistent across sexes.

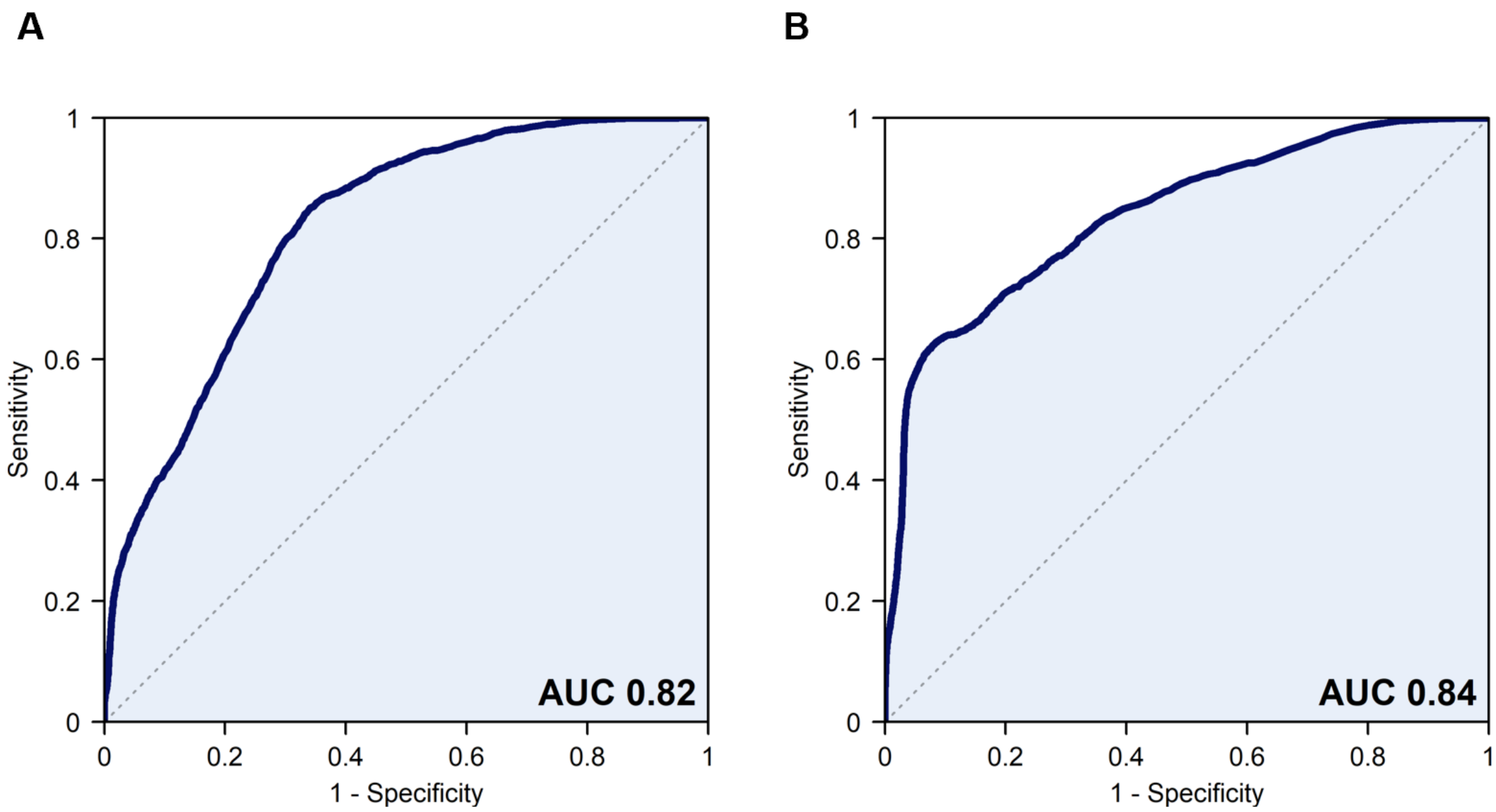

SI Figure 5 ROC curves for sex-stratified models. Comparable performance across sexes indicates that the classification is not driven by sex-specific effects. **A)** Male cohort; **B)** Female cohort. ROC, receiver-operating characteristic; AUC, area under the curve.

Assessment of sex as a potential confounder

SI Table 6 Two-way ANOVA assessing the effects of sex (and cancer) on top-ranked features. P-values are reported for the main effects of sex and cancer status, as well as their interaction. False discovery rate (FDR)-adjusted p-values are provided to account for multiple testing.

Variable	p Sex	p Interaction	FDR Sex	FDR Interaction
Lymphocyte count	0.6759	0.3780	0.7663	0.9642
HDL-p	0.0006	0.7189	0.0061	0.9642
SHDL-p	0.0418	0.7283	0.0996	0.9642
Mobile -CH_3 HDL/LDL	0.0010	0.9025	0.0061	0.6483
3-Hydroxybutyrate	0.1474	0.8491	0.2512	0.9642
Glutamate	0.0929	0.0291	0.1716	0.3010
Glutamate/Glutamine	0.0024	0.0122	0.0106	0.1887
Glutamine	0.0023	0.0003	0.0106	0.0098
Myo-inositol	0.4209	0.6136	0.5362	0.9642
Myo-inositol	0.0006	0.1046	0.0061	0.6483
Histidine	0.1540	0.9642	0.2512	0.9642
Histidine	0.9605	0.3871	0.9605	0.9642
FA1G1S1	0.0014	0.5173	0.0090	0.9642
FA2BG1	0.0225	0.8277	0.0698	0.9642
M4A1G1S1	0.0941	0.8686	0.1716	0.9642
FA2G2	0.0395	0.4552	0.0996	0.9642
FA2G2S1	0.0733	0.9465	0.1598	0.9642
FA2BG1	0.0122	0.4802	0.0473	0.9642
FA2G2S2	0.0010	0.7915	0.0079	0.9642
FA2BG2	0.0250	0.6106	0.0704	0.9642
M5A1G1	0.4497	0.5234	0.5362	0.9642
M5A1G1S1	0.0141	0.9573	0.0485	0.9642
M5A1G1S1	0.4388	0.4392	0.5362	0.9642
FA3G3S3	0.0773	0.5687	0.1598	0.9642
Cluster 22.4 min (FA1G1)	0.1741	0.3460	0.2621	0.9642
Cluster 23.4 min (FA1G1)	0.3314	0.7363	0.4467	0.9642
Cluster 25.9 min (M5A1G1)	0.2468	0.6876	0.3478	0.9642
Cluster 31.3 min (FA2G2S2)	0.6921	0.1846	0.7663	0.9539
Cluster 40.2 min (A3G3S2)	0.7869	0.0740	0.8412	0.5732
Cluster 40.3 min (A3G3S3)	0.1776	0.7070	0.2621	0.9642

Assessment of age as a potential confounder

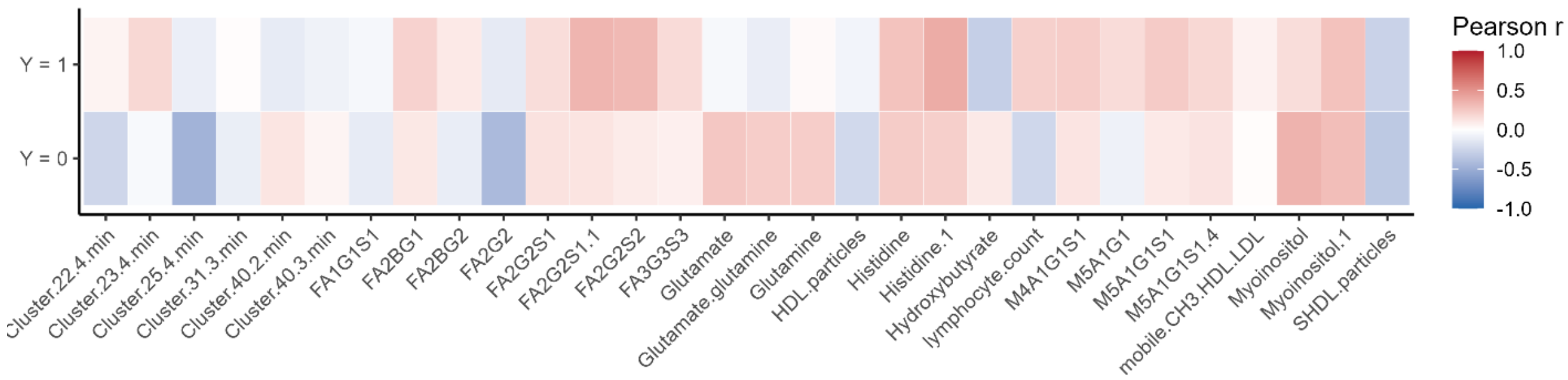

SI Figure 6 Assessment of age as a potential confounder of model-derived features. Heatmap showing Pearson correlation coefficients between age at SCAN and the highest-ranked metabolic and glycomic variables contributing to the cancer classification model, stratified by outcome group (Y = 1, cancer; Y = 0, non-cancer). Colour intensity reflects the direction and magnitude of correlation (red, positive; blue, negative). Across both outcome groups, correlations between age and model-informative variables were uniformly weak ($|r| < 0.35$), indicating that model discrimination was not driven by age-related effects. HDL-p, high-density lipoprotein particle concentration; SHDL-p, small high-density lipoprotein particle concentration. Glycan nomenclature follows the Oxford glycan notation described in the Methods section.

Assessment of age as a potential confounder

To assess the potential influence of age, the primary classification model was re-run with age included as a covariate. Model performance remained comparable to the original analysis, indicating that the discriminatory signal is not driven by age.

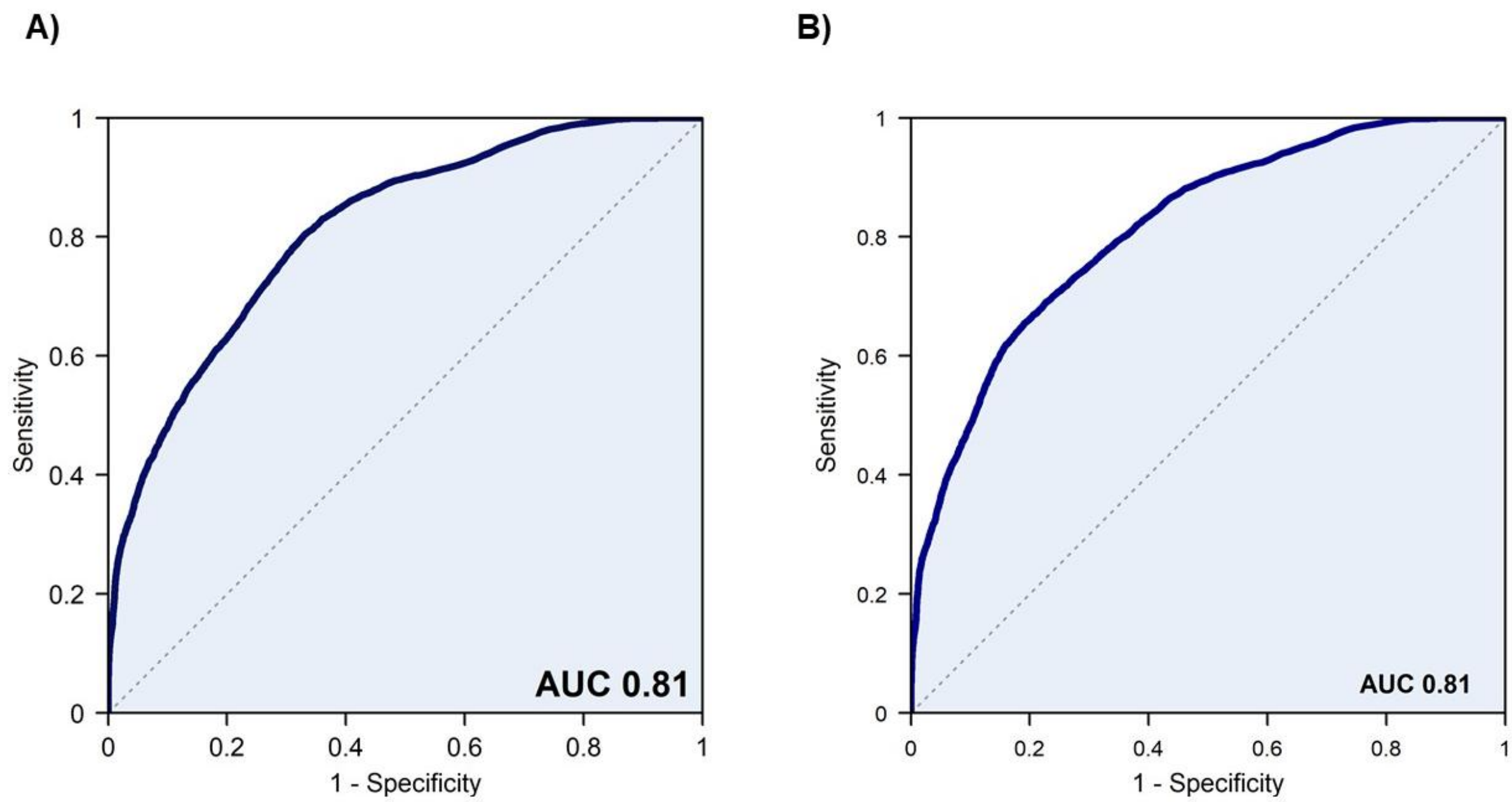

SI Figure 7 Effect of age inclusion on model discrimination in the SCAN2 cohort. ROC curves showing model performance when all variables, including age, are included **(A)** and when age is excluded **(B)**. Both models demonstrate identical discrimination (AUC 0.814), indicating that inclusion of age does not materially influence overall model performance. ROC, receiver-operating characteristic; AUC, area under the curve.

Assessment of age as a potential confounder

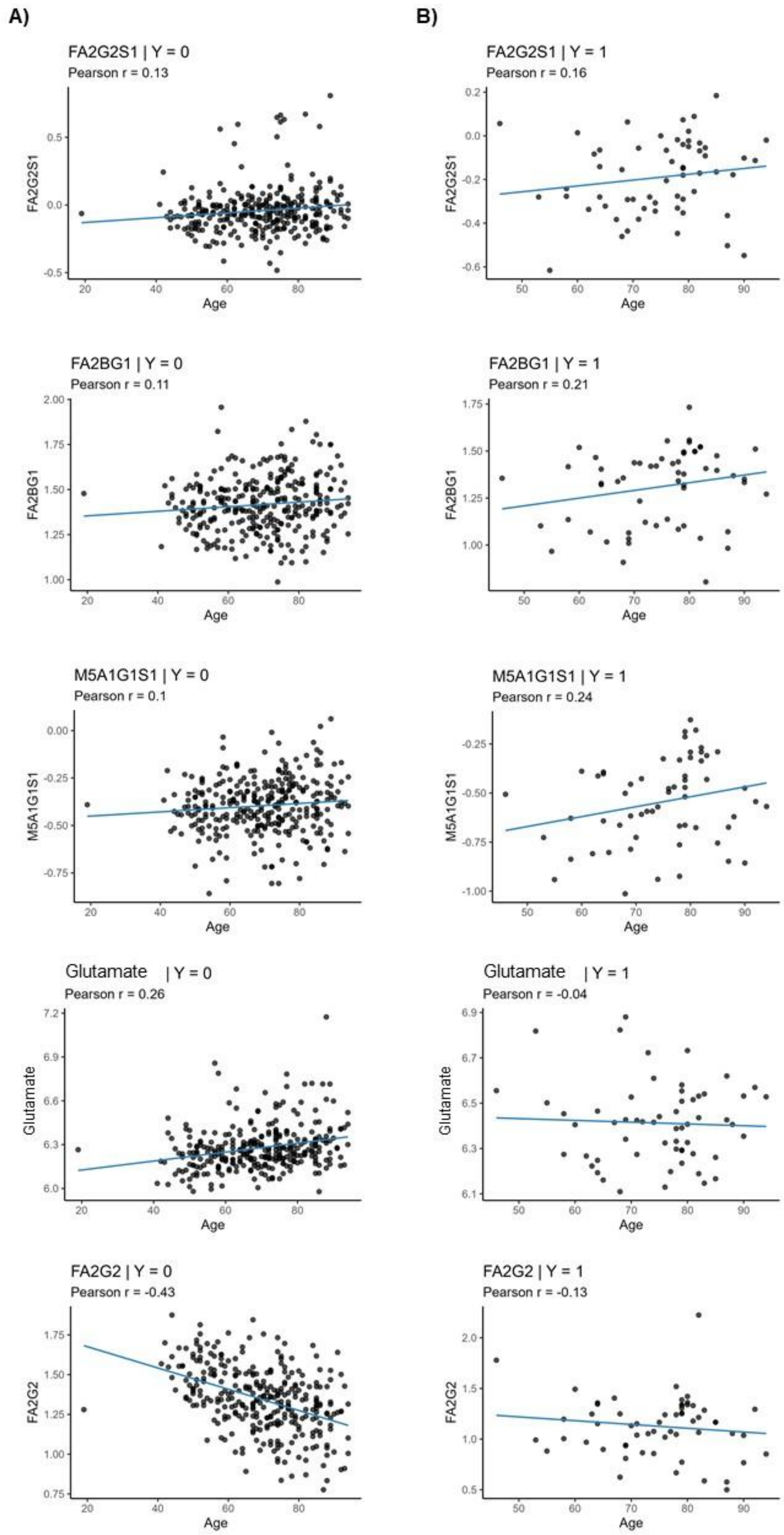

SI Figure 8 Representative Pearson correlation analyses between age and model-informative features. Scatter plots illustrate the relationship between age at SCAN and selected high-ranking variables from the cancer classification model, shown separately for non-cancer (Y = 0, (**A**)) and cancer (Y = 1, (**B**)) participants. Solid lines denote linear fits, with Pearson correlation coefficients indicated in each panel. Across both outcome groups, correlations were weak and inconsistent in direction, supporting minimal age dependence of individual model features.

Assessment of age as a potential confounder

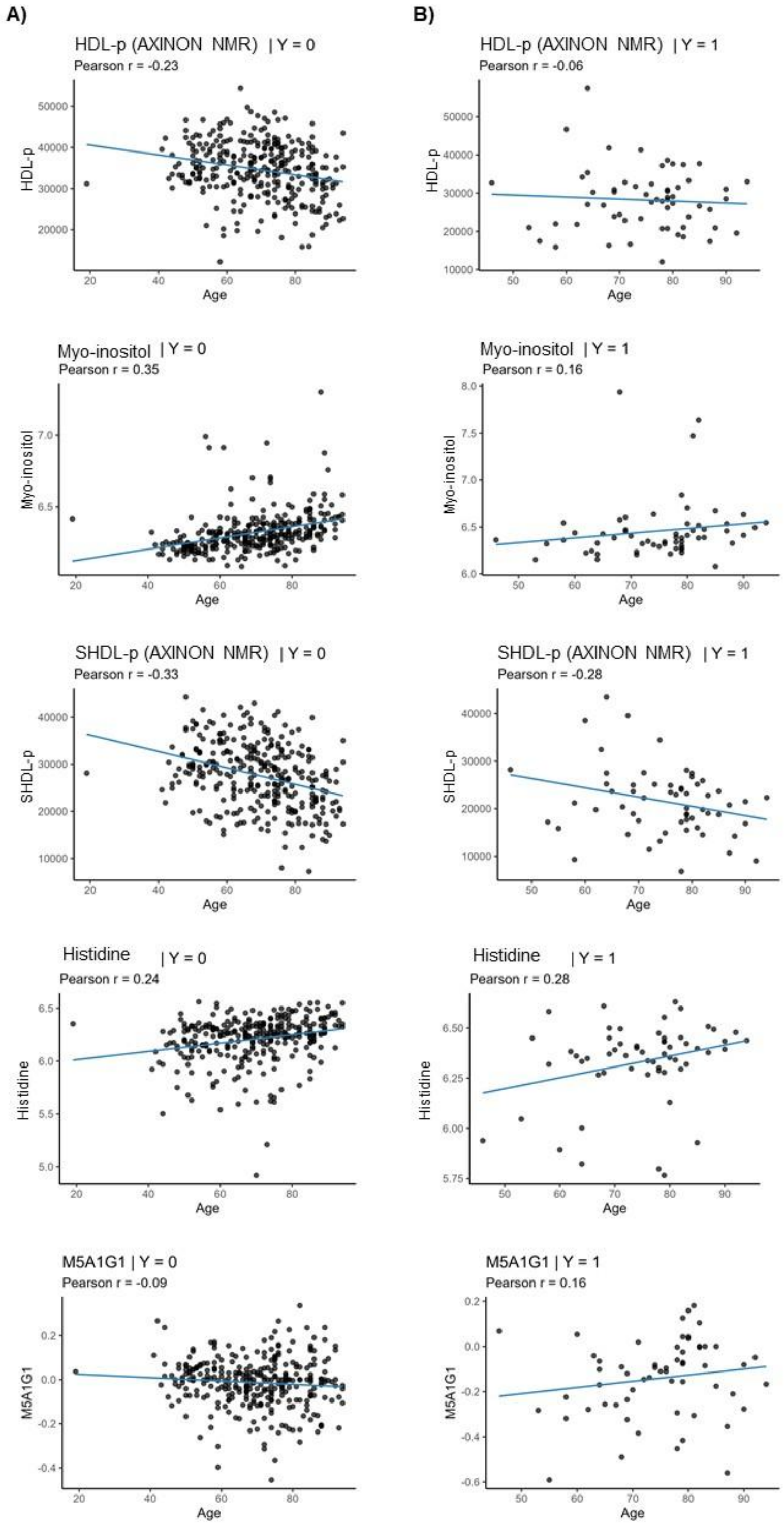

SI Figure 9 Representative Pearson correlation analyses between age and model-informative features. Scatter plots illustrate the relationship between age at SCAN and selected high-ranking variables from the cancer classification model, shown separately for non-cancer (Y = 0, (**A**)) and cancer (Y = 1, (**B**)) participants. Solid lines denote linear fits, with Pearson correlation coefficients indicated in each panel. Across both outcome groups, correlations were weak and inconsistent in direction, supporting minimal age dependence of individual model features.

Assessment of age as a potential confounder

To further evaluate the potential confounding effect of age on model performance, an age-matched subset of participants was constructed in which cancer and non-cancer groups were closely matched for age. Demographic characteristics of this subset are summarised below. Classification performance was assessed using the same modelling framework as in the primary analysis.

SI Table 7 Demographic characteristics of the age-matched subset used to assess the potential confounding effect of age.

Characteristics	Non-cancer	Cancer	*p*-value
n (participants)	22	34	
Age at SCAN	74.8 ± 10.4	74.8 ± 10.4	0.999
Sex (% female)	12 (55%)	17 (50%)	0.790
Body Mass Index, kg m^{-2}	25.2 ± 4.4	25.7 ± 5.6	0.704

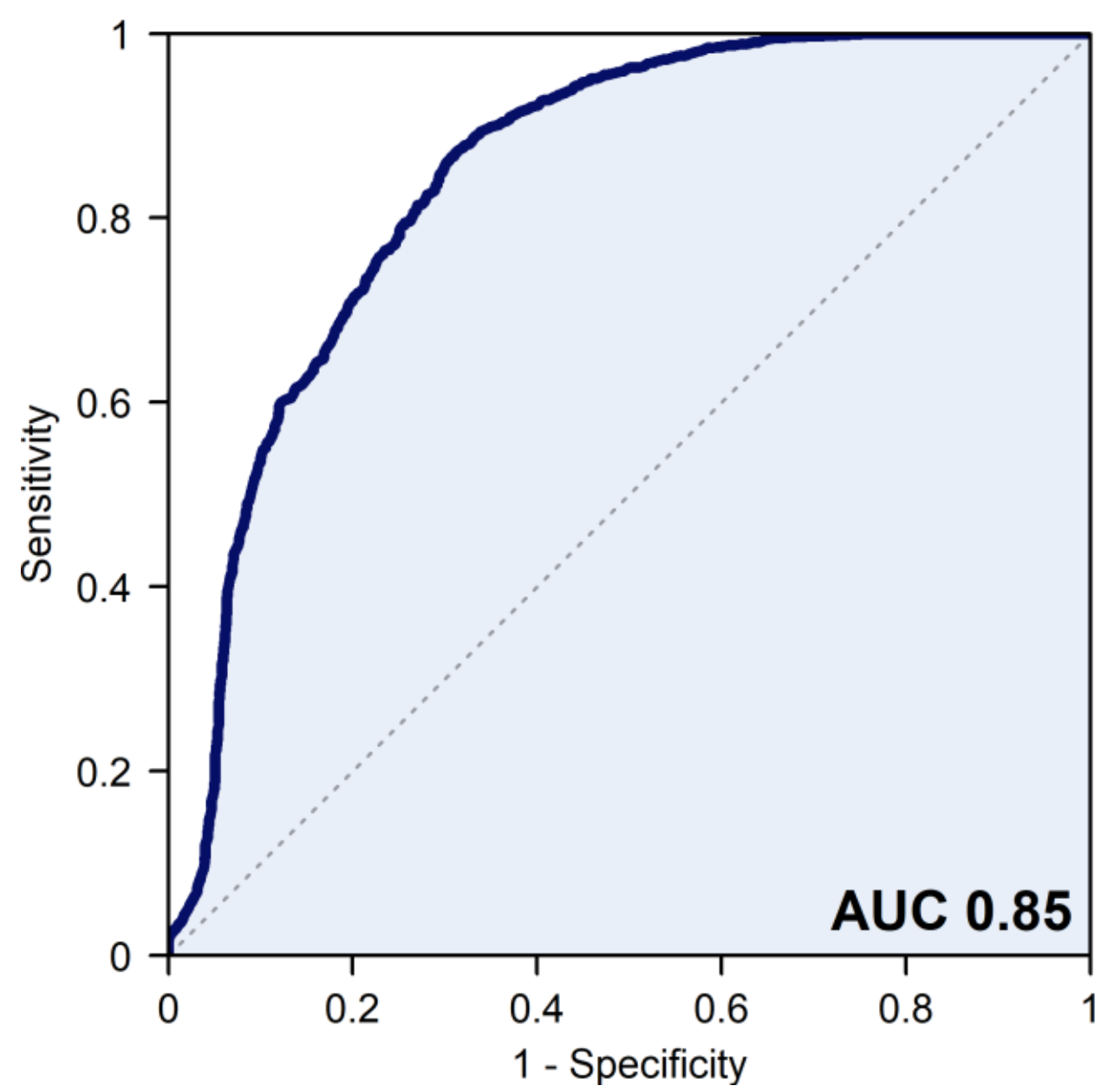

SI Figure 10 Effect of age matching on model discrimination in the SCAN2 cohort. ROC curve for the age-matched subset, in which cancer and non-cancer groups were matched for age to assess the potential confounding effect of age on model performance. The model includes all variables, including age. ROC, receiver-operating characteristic; AUC, area under the curve.

AUC 0.846 (95% CI 0.834-0.859)

Accuracy: 80.9% (95% CI 79.9-81.9)

Sensitivity: 86.6% (95% CI 85.7-87.6)

Specificity: 70.3% (95% CI 69.5-71.1)

Association of renal function with cancer status

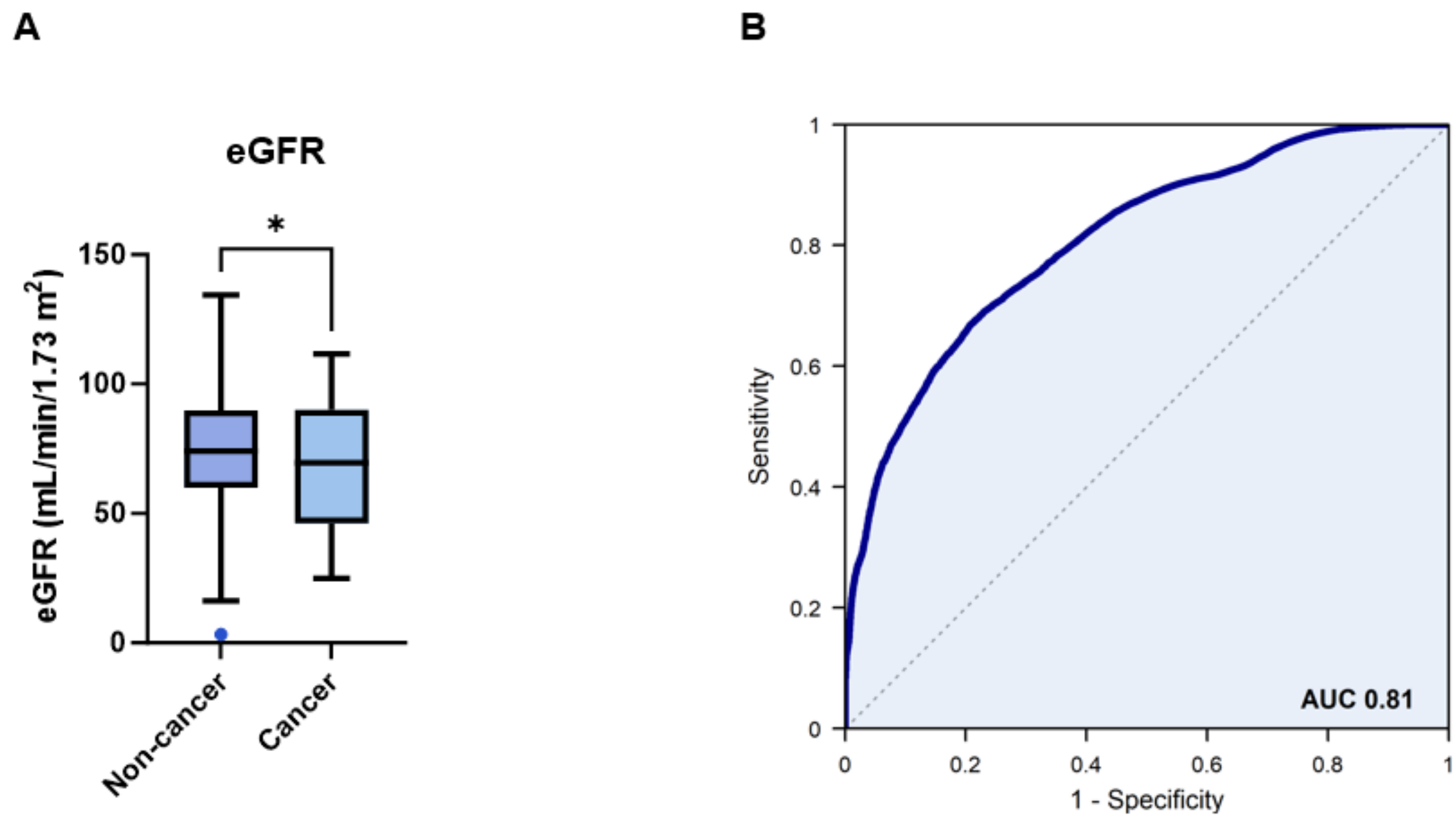

SI Figure 11 Association of renal function with cancer status and discriminatory performance. (A) Creatinine-derived estimated glomerular filtration rate (eGFR) in cancer and non-cancer participants (p = 0.013). Boxes indicate median and interquartile range; whiskers denote Tukey limits. **(B)** ROC curves showing model performance with all variables, including eGFR (ROC AUC 0.809 (95% CI 0.803-0.815)). Despite a modest univariable association, eGFR was not prioritised in multivariable feature selection and did not materially influence model performance. ROC, receiver-operating characteristic; AUC, area under the curve; eGFR, estimated glomerular filtration rate.

Validation in the SCAN1 Cohort

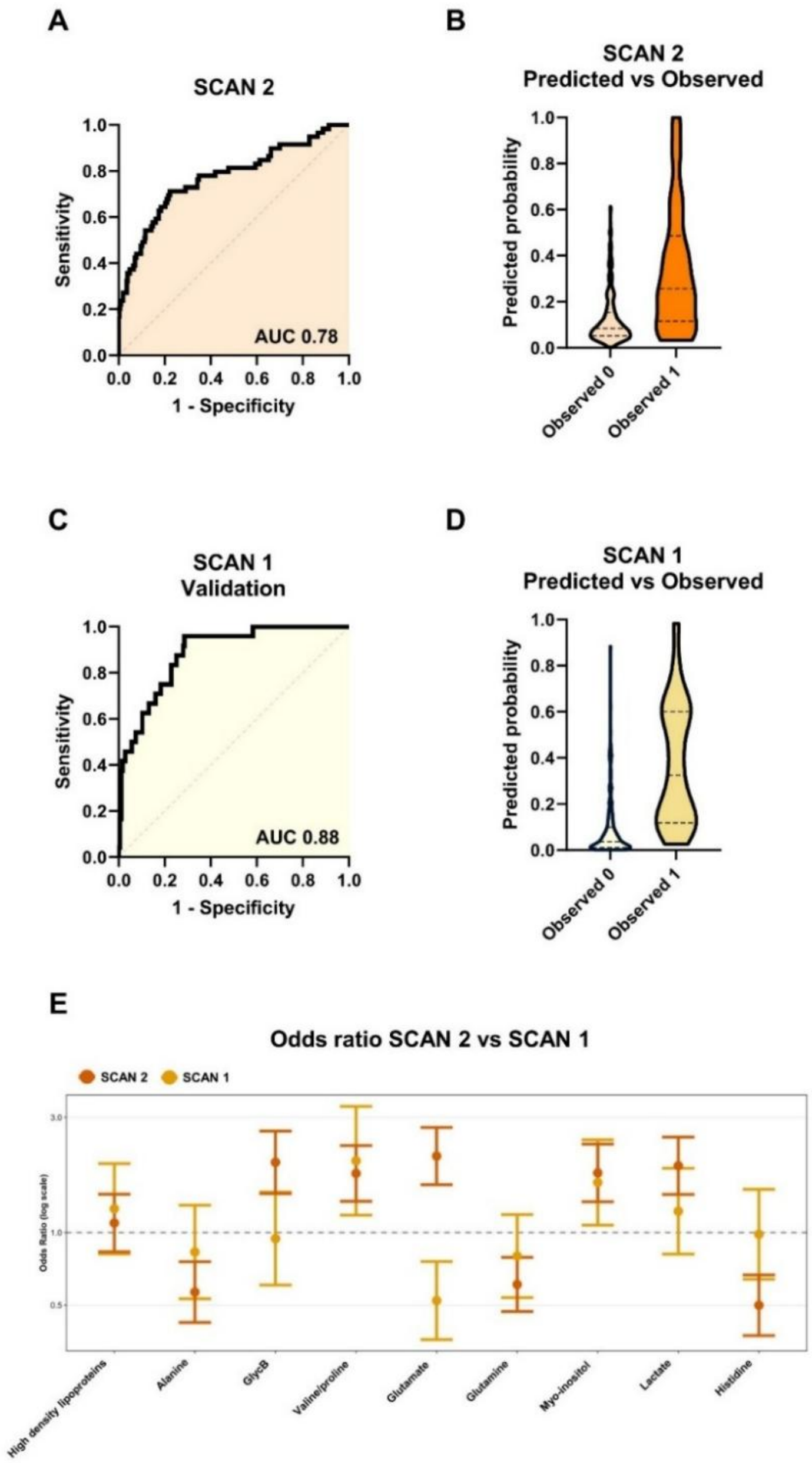

SI Figure 12 External validation of the nine most discriminatory NMR-derived metabolites between SCAN2 and SCAN1 samples. (A) ROC curve for the SCAN2 model built using the nine top-ranked metabolites (glutamate, histidine, myo-inositol, glycine, valine, leucine, isoleucine, HDL-p, SHDL-p), yielding an AUC 0.783 (95% CI 0.774-0.791). **(B)** Predicted versus observed probabilities for cancer and non-cancer cases in SCAN2, showing clear separation between groups. **(C)** ROC curve for validation in SCAN1 using the same nine metabolites, demonstrating higher discrimination (AUC 0.881 (95% CI 0.875-0.896)). **(D)** Predicted versus observed probabilities in SCAN1, confirming reproducible classification performance across cohorts. **(E)** Comparison of ORs (log scale, ±95% CI) for the nine metabolites in SCAN2 and SCAN1, showing consistent effect directions: amino-acid metabolites exhibited higher odds of cancer, whereas HDL-related measures were associated with lower odds in both cohorts. ROC, receiver-operating characteristic; AUC, area under the curve. HDL-p, high-density lipoprotein particle concentration; SHDL-p, small high-density lipoprotein particle concentration; OR, odds ratio.

Discrimination between cancer and non-cancer individuals using NMR metabolomics and LC-MS glycomics

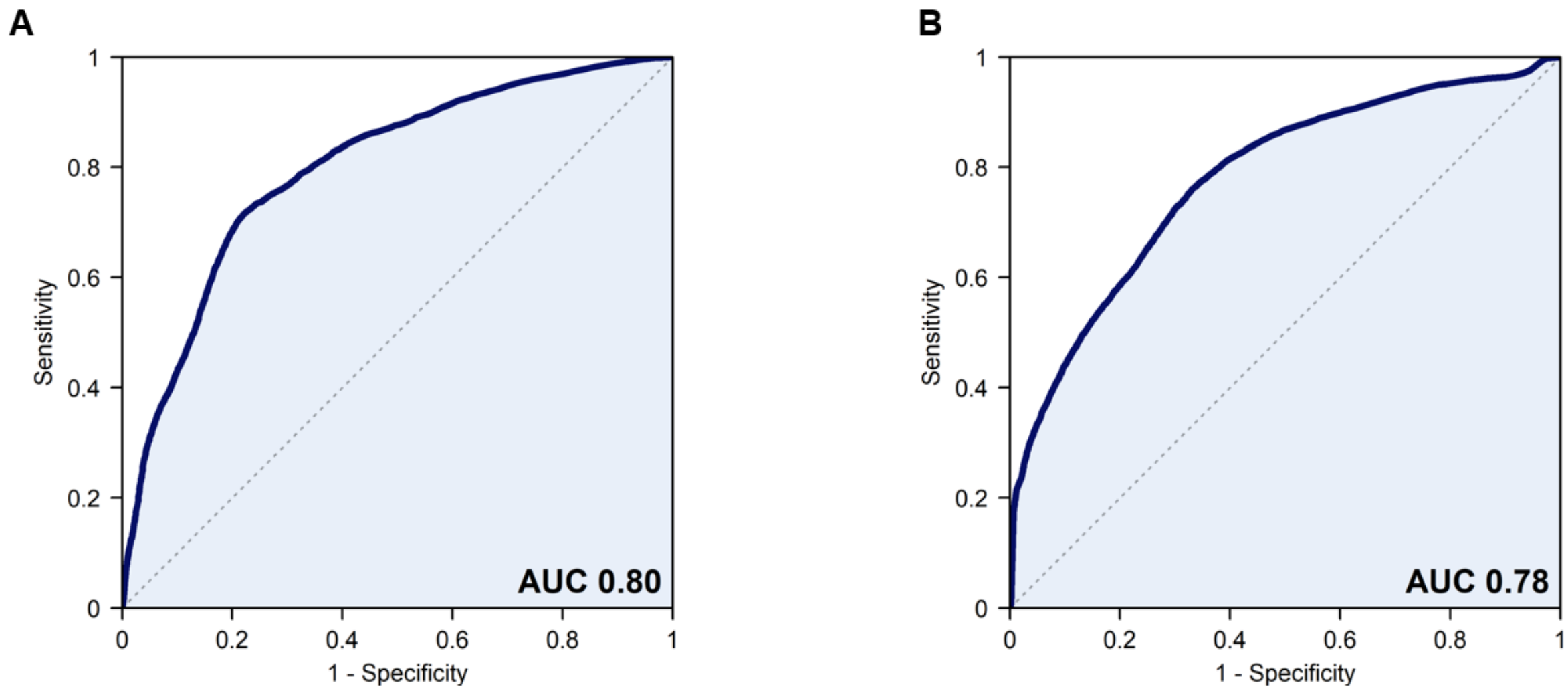

SI Figure 13 Diagnostic performance of individual analytical platforms for distinguishing cancer from non-cancer in the SCAN2 cohort (*n* = 369; 59 cancer, 310 non-cancer). **(A)** ROC curve for the NMR model integrating untargeted metabolomics and targeted *AXINON® System* clinical parameters. The model achieved an AUC 0.799 (95% CI 0.793-0.805), demonstrating strong discriminatory capacity between cancer and non-cancer cases. **(B)** ROC curve for the semi-targeted glycomics model combining fluorescence and MS-based data, which achieved an AUC 0.778 (95% CI 0.771-0.785), indicating moderate but consistent discrimination across the same cohort. Both models were derived using random forest classifiers with stratified cross-validation and validated in the full SCAN2 dataset. ROC, receiver-operating characteristic; AUC, area under the curve.

SI Table 8 Top discriminatory variables distinguishing cancer from non-cancer cases. Variables were selected from the integrated random-forest model including all participants (*n* = 369; 59 cancer, 310 non-cancer) and are ranked by Mean Decrease Gini index. Odds ratios (OR) with 95% confidence intervals quantify the direction and strength of association with cancer. Statistical significance was assessed using two-sided *t*-tests, with *p*-values corrected for multiple testing using the Benjamini–Hochberg method. For untargeted ^{1}H NMR data, reported chemical shifts correspond to the beginning of each 0.01 ppm spectral bin. Fluorescence-derived glycan clusters correspond to retention-time peaks, with the predominant glycan structure indicated for each cluster. Adjusted p-values are annotated as follows: ns, not significant ($p \geq 0.05$); $p < 0.05$ (*); $p < 0.01$ (**); $p < 0.001$ (***); and $p < 0.0001$ (****).

Variable	Platform	Mean Decrease Gini	OR	Lower 95% CI	Upper 95% CI	p-value (raw)	p-value (adj.)	Significance
FA2G2S1	Glycan (MS)	2.827	0.299	0.200	0.448	4.68E-08	7.81E-07	****
FA2BG1	Glycan (MS)	1.876	0.514	0.386	0.685	2.35E-04	4.15E-04	***
M5A1G1S1	Glycan (MS)	1.570	0.406	0.298	0.553	2.28E-06	9.77E-06	****
Glutamate (2.36 ppm)	NMR	1.424	2.075	1.579	2.726	5.40E-07	3.63E-06	****
M4A1G1S1	Glycan (MS)	1.400	0.433	0.320	0.586	8.01E-06	2.40E-05	****
FA2G2	Glycan (MS)	1.170	0.379	0.278	0.516	7.26E-07	3.63E-06	****
HDL-p	NMR (AXINON)	1.115	0.435	0.328	0.576	3.35E-07	3.35E-06	****
M5A1G1S1-4	Glycan (MS)	1.101	0.713	0.533	0.954	8.46E-02	9.76E-02	ns
Myo-inositol (3.62 ppm)	NMR	1.062	1.767	1.342	2.327	2.21E-03	3.01E-03	**
SHDL-p	NMR (AXINON)	1.034	0.435	0.324	0.582	5.21E-08	7.81E-07	****
Histidine (7.04 ppm)	NMR	1.027	2.441	1.562	3.817	3.69E-05	8.51E-05	****
M5A1G1	Glycan (MS)	1.015	0.397	0.294	0.537	6.13E-07	3.63E-06	****
FA2G2S1	Glycan (MS)	0.997	0.299	0.200	0.448	6.23E-05	1.34E-04	****
FA2G2S2	Glycan (MS)	0.977	0.564	0.439	0.724	1.55E-04	3.10E-04	***
FA2BG2	Glycan (MS)	0.933	0.524	0.398	0.691	1.90E-04	3.56E-04	***
Cluster 25.9 min (M5A1G1)	Glycan (fluorescence)	0.931	0.415	0.307	0.559	4.90E-06	1.77E-05	****
Histidine (7.78 ppm)	NMR	0.930	1.905	1.377	2.635	3.77E-04	6.28E-04	***
Cluster 40.2 min (A3G3S2)	Glycan (fluorescence)	0.904	1.653	1.235	2.213	6.16E-03	7.70E-03	**
Mobile -CH_3 HDL/LDL (0.83 ppm)	NMR	0.893	0.817	0.615	1.085	2.31E-01	2.48E-01	ns
glutamine/glutamate (2.38 ppm)	NMR	0.881	2.082	1.503	2.884	8.80E-04	1.39E-03	**
Cluster 40.3 min (A3G3S3)	Glycan (fluorescence)	0.881	2.062	1.552	2.739	1.07E-05	2.67E-05	****
Myo-inositol (4.04 ppm)	NMR	0.880	1.412	1.082	1.843	7.91E-02	9.50E-02	ns
FA3G3S3	Glycan (MS)	0.872	0.432	0.313	0.595	5.30E-06	1.77E-05	****
Cluster 22.4 min (FA1G1)	Glycan (fluorescence)	0.834	0.485	0.363	0.649	1.01E-03	1.51E-03	**
Cluster 23.4 min (FA1G1)	Glycan (fluorescence)	0.831	0.550	0.416	0.728	1.06E-03	1.51E-03	**
Cluster 31.3 min (FA2G2S2)	Glycan (fluorescence)	0.820	0.649	0.472	0.891	1.33E-05	1.88E-05	****
Lymphocyte count	Haematology	0.818	0.739	0.552	0.990	7.14E-01	7.14E-01	ns

Glutamine (2.39 ppm)	NMR	0.817	2.014	1.534	2.644	9.52E-06	2.60E-05	****
3-Hydroxybutyrate (1.18 ppm)	NMR	0.817	1.590	1.236	2.045	2.96E-03	3.86E-03	**
FA1G1S1	Glycan (MS)	0.812	0.851	0.640	1.130	2.69E-01	2.78E-01	ns

OR, odds ratio; HDL-p, high-density lipoprotein particle concentration; SHDL-p, small high-density lipoprotein particle concentration. Glycan nomenclature follows the Oxford glycan notation described in the Methods section.

SI Table 9 Top discriminatory variables distinguishing cancer from non-cancer cases (selected sub-set based on cancer type and comorbidities). Variables were selected from the integrated random-forest model including selected participants (n = 309; 32 cancer, 277 non-cancer) and are ranked by Mean Decrease Gini index. Odds ratios (OR) with 95% confidence intervals quantify the direction and strength of association with cancer. Statistical significance was assessed using two-sided t-tests, with p-values corrected for multiple testing using the Benjamini–Hochberg method. For untargeted ^{1}H NMR data, reported chemical shifts correspond to the beginning of each 0.01 ppm spectral bin. Fluorescence-derived glycan clusters correspond to retention-time peaks, with the predominant glycan structure indicated for each cluster. Adjusted p-values are annotated as follows: ns, not significant ($p \geq 0.05$); $p < 0.05$ (*); $p < 0.01$ (**); $p < 0.001$ (***); and $p < 0.0001$ (****).

Variable	Platform	Mean Decrease Gini	OR	Lower 95% CI	Upper 95% CI	p-value (raw)	p-value (adj.)	Significance
FA2G2S1	Glycan (MS)	1.450	0.241	0.140	0.414	1.86E-05	9.29E-05	****
M4A1G1S1	Glycan (MS)	1.157	0.350	0.237	0.518	8.56E-05	2.85E-04	***
M5A1G1S1	Glycan (MS)	1.076	0.296	0.194	0.451	6.68E-06	5.01E-05	****
Cluster 28.1 min (FA2BG1S1)	Glycan (fluorescence)	0.909	0.390	0.263	0.580	2.87E-04	6.15E-04	***
FA2BG1	Glycan (MS)	0.845	0.461	0.316	0.671	1.63E-03	2.99E-03	**
Glutamine (2.39 ppm)	NMR	0.801	2.568	1.815	3.634	1.28E-04	3.24E-04	***
Glutamate (2.36 ppm)	NMR	0.781	2.680	1.890	3.801	3.55E-06	3.55E-05	****
FA2G2	Glycan (MS)	0.773	0.281	0.183	0.430	4.40E-07	6.60E-06	****
Myo-inositol (4.04 ppm)	NMR	0.727	1.323	0.992	1.765	3.06E-01	3.40E-01	ns
Glutamine/glutamate (2.38 ppm)	NMR	0.709	2.998	1.927	4.665	2.58E-03	4.07E-03	**
Leucine (0.97 ppm)	AXINON	0.681	0.625	0.421	0.928	3.56E-02	4.45E-02	*
M5A1G1	Glycan (MS)	0.668	0.332	0.227	0.488	1.82E-05	9.29E-05	****
Cluster 25.9 min (M5A1G1)	Glycan (fluorescence)	0.664	0.290	0.192	0.440	2.51E-07	6.60E-06	****
M5A1G1S1-4	Glycan (MS)	0.638	0.677	0.460	0.996	2.01E-01	2.32E-01	ns
Glutamate (2.37 ppm)	NMR	0.630	2.328	1.681	3.225	1.40E-04	3.24E-04	***
Cluster 40.2 min (A3G3S2)	Glycan (fluorescence)	0.619	1.762	1.210	2.567	3.21E-02	4.19E-02	*
Cluster 35.2 min (complex glycan)	Glycan (fluorescence)	0.617	0.972	0.674	1.401	9.68E-01	9.68E-01	ns
Myo-inositol (3.62 ppm)	NMR	0.538	2.509	1.612	3.905	4.46E-03	6.37E-03	**
Formate (8.45 ppm)	NMR	0.538	1.038	0.710	1.516	8.50E-01	8.79E-01	ns
Cluster 31.3 min (FA2G2S2)	Glycan (fluorescence)	0.538	0.680	0.451	1.024	4.91E-01	5.26E-01	ns
FA1G1S1	Glycan (MS)	0.519	0.741	0.508	1.080	1.49E-01	1.78E-01	ns
Cluster 31.7 min (A2G2S2)	Glycan (fluorescence)	0.512	0.406	0.266	0.619	4.09E-04	8.18E-04	***
Lactate (4.13 ppm)	NMR	0.491	2.176	1.553	3.048	1.69E-03	2.99E-03	**
Cluster 23.4 min (FA1G1)	Glycan (fluorescence)	0.490	0.447	0.311	0.643	1.83E-03	3.05E-03	**

A1G1S1	Glycan (MS)	0.489	0.552	0.376	0.811	2.10E-02	2.86E-02	*
FA2G2S1	Glycan (MS)	0.482	0.407	0.292	0.569	1.31E-04	3.24E-04	***
HDL-p	AXINON	0.467	0.421	0.300	0.589	1.16E-04	3.24E-04	***
Valine	AXINON	0.459	0.427	0.283	0.642	6.69E-05	2.51E-04	***
Histidine (7.78 ppm)	NMR	0.441	2.486	1.389	4.450	3.36E-03	5.03E-03	**
Cluster 41.5 min (A4G4S4)	Glycan (fluorescence)	0.440	2.416	1.692	3.448	5.85E-05	2.51E-04	***

OR, odds ratio; HDL-p, high-density lipoprotein particle concentration; SHDL-p, small high-density lipoprotein particle concentration. Glycan nomenclature follows the Oxford glycan notation described in the Methods section.

Sensitivity Analysis of the Dual-Probability Classification Framework

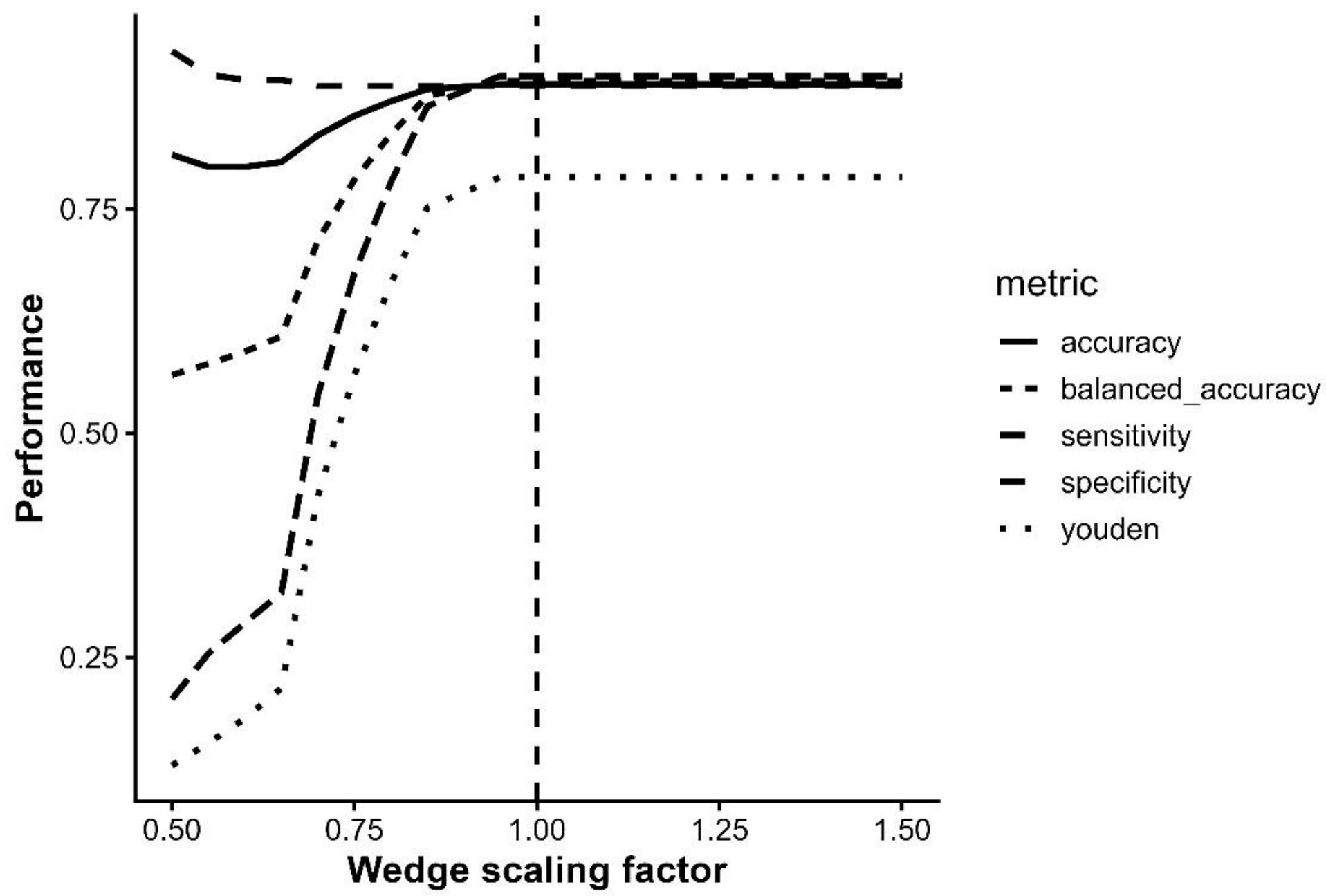

SI Figure 14 Sensitivity analysis of the KDE-derived wedge prediction region. The wedge-shaped prediction region used to identify individuals within the overlap between cancer and alternative diagnostic groups was derived from the kernel density estimation (KDE) analysis. To evaluate the robustness of this approach, the wedge geometry was systematically varied, and more than 20,000 candidate configurations were tested. Classification performance was assessed using accuracy, balanced accuracy, sensitivity, specificity, and Youden's index. The vertical dashed line indicates the wedge used in the main analysis (scaling factor = 1.0). Performance metrics reached a plateau close to the selected wedge dimensions and remained largely unchanged across a broad range of scaling factors, indicating that the results were not sensitive to the precise choice of wedge geometry.

SI Table 10 Top discriminatory variables distinguishing metastatic from non-metastatic cancer. Variables were selected from the integrated random-forest model including all participants (*n* = 59; 29 metastatic cancer, 30 non-metastatic cancer) and are ranked by Mean Decrease Gini index. Odds ratios (OR) with 95% confidence intervals quantify the direction and strength of association with metastatic disease. Statistical significance was assessed using two-sided *t*-tests, with *p*-values corrected for multiple testing using the Benjamini–Hochberg method. For untargeted ^{1}H NMR data, reported chemical shifts correspond to the beginning of each 0.01 ppm spectral bin. Fluorescence-derived glycan clusters correspond to retention-time peaks, with the predominant glycan structure indicated for each cluster. Adjusted p-values are annotated as follows: ns, not significant (p ≥ 0.05); p < 0.05 (*); p < 0.01 (**); p < 0.001 (***); and p < 0.0001 (****).

Variable	Platform	Mean Decrease Gini	OR	Lower 95% CI	Upper 95% CI	p-value (raw)	p-value (adj.)	Significance
Histidine (7.79 ppm)	NMR	0.629	0.413	0.211	0.809	0.004	0.018	*
FA4G4S4	Glycan (MS)	0.521	2.325	1.250	4.327	0.004	0.018	*
Histidine (7.04 ppm)	NMR	0.481	0.300	0.127	0.713	0.002	0.015	*
Alanine (1.47 ppm)	NMR	0.480	0.387	0.195	0.767	0.002	0.015	*
Alanine (1.46 ppm)	NMR	0.474	0.384	0.193	0.764	0.002	0.015	*
Cluster 31.7 min	Glycan (fluorescence)	0.429	0.287	0.119	0.695	0.002	0.015	*
Alanine	NMR (AXINON)	0.403	0.362	0.180	0.729	0.002	0.015	*
Myo-inositol (3.63 ppm)	NMR	0.394	1.399	0.774	2.531	0.270	0.338	ns
Cluster 40.8 min	Glycan (fluorescence)	0.372	1.940	1.064	3.539	0.022	0.050	ns
Cluster 33.9 min	Glycan (fluorescence)	0.333	0.482	0.260	0.894	0.015	0.042	*
Myo-inositol (3.62 ppm)	NMR	0.322	1.241	0.710	2.168	0.472	0.488	ns
Free choline/-N(CH3)3 (3.21 ppm)	NMR	0.291	0.668	0.388	1.150	0.153	0.219	ns
Glucose region (3.86 ppm)	NMR	0.281	1.430	0.805	2.539	0.222	0.290	ns
Myo-inositol (3.64 ppm)	NMR	0.276	1.529	0.811	2.882	0.176	0.240	ns
Cluster 23.8 min	Glycan (fluorescence)	0.274	0.584	0.328	1.038	0.068	0.128	ns
Cluster 24.4 min	Glycan (fluorescence)	0.265	0.472	0.256	0.869	0.010	0.030	*
FA4G4S4	Glycan (MS)	0.260	4.200	1.383	12.758	0.007	0.024	*
Isoleucine (0.93 ppm)	NMR	0.254	0.653	0.370	1.150	0.137	0.216	ns
Mobile -CH3 HDLs (0.84 ppm)	NMR	0.252	1.498	0.855	2.623	0.154	0.219	ns
Myo-inositol (3.65 ppm)	NMR	0.251	1.365	0.754	2.470	0.307	0.368	ns
Glutamine (2.42 ppm)	NMR	0.248	0.595	0.340	1.040	0.060	0.121	ns
FA3G3S1	Glycan (MS)	0.247	1.632	0.915	2.909	0.087	0.154	ns

A4G4S2	Glycan (MS)	0.242	1.203	0.713	2.030	0.574	0.574	ns
Cluster 25.9 min	Glycan (fluorescence)	0.235	0.436	0.227	0.838	0.008	0.027	*
Myo-inositol (3.66 ppm)	NMR	0.234	1.275	0.723	2.247	0.408	0.447	ns
M4A1G1S1	Glycan (MS)	0.230	1.226	0.727	2.067	0.417	0.447	ns
Cluster 39.0 min	Glycan (fluorescence)	0.228	1.741	0.902	3.361	0.095	0.159	ns
Citrate (2.67 ppm)	NMR	0.225	1.256	0.744	2.120	0.382	0.441	ns
FA4FG4S4	Glycan (MS)	0.224	2.058	1.129	3.753	0.018	0.045	*
Glucose region (3.75 ppm)	NMR	0.221	0.571	0.314	1.040	0.057	0.121	ns

OR, odds ratio; HDL-p, high-density lipoprotein particle concentration; SHDL-p, small high-density lipoprotein particle concentration. Glycan nomenclature follows the Oxford glycan notation described in the Methods section.
